# Quantifier processing and semantic flexibility in patients with aphasia

**DOI:** 10.3389/fpsyg.2024.1328853

**Published:** 2024-07-18

**Authors:** Birte Reißner, Wiebke Grohmann, Natalja Peiseler, João Pinho, Katja Hußmann, Cornelius J. Werner, Stefan Heim

**Affiliations:** ^1^Department of Neurology, Medical Faculty, RWTH Aachen University, Aachen, Germany; ^2^Department of Psychiatry, Psychotherapy and Psychosomatics, Medical Faculty, RWTH Aachen University, Aachen, Germany; ^3^Department of Linguistics, Heinrich Heine University, Düsseldorf, Germany; ^4^Johanniter Hospital Stendal, Stendal, Germany; ^5^Institute of Neuroscience and Medicine (INM-1), Forschungszentrum Jülich GmbH, Jülich, Germany

**Keywords:** quantifier, semantics, aphasia, flexibility, adaptation, feedback, learning

## Abstract

Processing of quantifiers such as “many” and “few” relies on number knowledge, linguistic abilities, and working memory. Negative quantifiers (e.g., “few,” “less than half”) induce higher processing costs than their positive counterparts. Furthermore, the meaning of some quantifiers is flexible and thus adaptable. Importantly, in neurotypical individuals, changing the meaning of one quantifier also leads to a generalized change in meaning for its polar opposite (e.g., the change of the meaning of “many” leads to the change of that of “few”). Here, we extended this research to patients with fluent and non-fluent aphasia after stroke. In two experiments, participants heard sentences of the type “Many/few of the circles are yellow/blue,” each followed by a picture with different quantities of blue and yellow circles. The participants judged whether the sentence adequately described the picture. Each experiment consisted of three blocks: a baseline block to assess the participants’ criteria for both quantifiers, a training block to shift the criteria for “many,” and a test block, identical to the baseline to capture any changes in quantifier semantics. In Experiment 1, the change of the meaning of “many” was induced by using adaptation to small numbers (20–50%) of circles of the named color. In Experiment 2, explicit feedback was given in the training block after each response to rate proportions of 40% (or higher) as “many,” whereas 40% is normally rather rated as “few.” The objective was to determine whether people with fluent or non-fluent aphasia were able to process quantifiers appropriately and whether generalized semantic flexibility was present after brain damage. Sixteen out of 21 patients were able to perform the task. People with fluent aphasia showed the expected polarity effect in the reaction times and shifted their criteria for “many” with generalization to the untrained quantifier “few.” This effect, however, was only obtained after explicit feedback (Experiment 2) but not by mere adaptation (Experiment 1). In contrast, people with non-fluent aphasia did not change the quantifier semantics in either experiment. This study contributes to gaining new insights into quantifier processing and semantic flexibility in people with aphasia and general underlying processing mechanisms.

## Introduction

### Quantifier processing

Quantifiers like “some,” “many,” “few,” “more than half” are words that describe quantities or proportions of sets and/or their relations. Having probably emerged in a process of cultural evolution (cf. Carcassi et al., 2021) they are a natural part of our everyday language and thinking. “I have worked for many hours today” or “I drank only a few cups of coffee” are examples of the numerous situations in which quantifiers are used. Quantifier processing requires number knowledge to assess quantities and their relations (Clark and Grossman, 2007; Ash et al., 2016) and language to grasp quantifiers linguistically and decode them semantically (Heim et al., 2012; Deschamps et al., 2015). Moreover, the semantic evaluation of quantifier statements requires working memory capacity (Caspari et al., 1998; Caplan and Waters, 1999, 2001; Wright and Shisler, 2005; Potagas et al., 2011; Mayer and Murray, 2012; Wright and Fergadiotis, 2012) for linking verbal to visuospatial and executive information (Baddeley, 2000, 2003). Previous studies indicated that quantifier processing differs depending on their polarity, i.e., whether they are positive or negative (Deschamps et al., 2015; Agmon et al., 2021; Grodzinsky et al., 2021): as indicated by longer reaction times (Agmon et al., 2019) processing of negative quantifiers (like “few,” “less,” which are monotone decreasing; cf. Barwise and Cooper, 1988) seems to be cognitively more demanding than processing of their positive counterparts (like “many,” “more,” which are monotone increasing) due to the additional negation they contain (“less than half” = “not more than half”). Among the word category of quantifiers, several sub-groups can be distinguished, e.g., cardinal (e.g., “five”; “at least seven”), majority (e.g., “most”), Aristotelean/logical (e.g., “all,” “some”), or parity quantifiers (e.g., “an odd number”), but also proportional quantifiers (e.g., “many”) (cf. McMillan et al., 2005; Clark and Grossman, 2007; Shikhare et al., 2015). Some types of quantifiers (e.g., majority quantifiers) are inherently more complex than either cardinal or Aristotelean quantifiers in regard to working memory demands because quantities must be counted or estimated and then memorized for comparison (Clark and Grossman, 2007).

Interestingly, the categorization of the quantifiers “many” and “few” (used in the present study) is not unequivocal. Assignment to the group of cardinal, proportional but also majority quantifiers, seems possible, depending on the context in which they are used (Bayırlı, 2022; cf. Oaksford et al., 2002; Pezzelle et al., 2018, for a proportional use of “few” when contrasted to a larger set of other quantifiers; but, e.g., Heim et al., 2015, for a majority-type of use when only “few” vs. “many” were used a quasi-polar opposites). One important characteristic of “many” and “few” is their semantic vagueness (Feiman and Snedeker, 2016; Pezzelle and Fernández, 2023). Barwise and Cooper (1988), borrowing the term from Milsark (1977; cited after Barwise and Cooper, 1988), distinguish them as “weak” quantifiers from “strong” quantifiers such as “all,” “every,” or “most.” In the context of the Generalized Quantifier Theory (Barwise and Cooper, 1988; von Fintel and Keenan, 2018; see their discussion on pp. 189–190), the weak proportional quantifiers “many” and “few” appear to violate the conservativity constraint, i.e., they may in some contexts be understood in their reverse (anti-proportional) meaning, thus resulting in a semantic variability or vagueness (see explanation and discussion in Bayırlı, 2022, see also Keenan and Stavi, 1986; Keenan, 2006; von Fintel and Keenan, 2018; Zuber and Keenan, 2019).

One aspect related to the” vagueness” of the quantifiers “few” and “many” is that their use is inherently dependent on variable internal criteria (Schöller and Franke, 2016, 2017; Heim et al., 2020b). For example, “many” cookies could mean three for one (full) person and 10 for another (hungry) one. These internal criteria are subjective and therefore depend on the individual (Ramotowska et al., 2023), but also on the object or subject to which a quantifier refers. This inter-subject variability is even further extended by a contextual variation (Schöller and Franke, 2017). The meaning of a quantifier varies depending on context in which the quantifier is used (Schöller and Franke, 2017). For example, “Few people celebrated Tom’s birthday” could mean five, whereas “Few people attended the World Cup” could refer to thousands. As previous experiments with neurotypical individuals show, these internal criteria for the preference of one quantifier over another can be changed by different learning contexts such as priming (Feiman and Snedeker, 2016), explicit reinforcement involving feedback (Heim et al., 2015, 2016, 2020a) or by adaptation (Helson, 1948; Heim et al., 2020b) and also as semantic alignment in active linguistic interaction (Pezzelle and Fernández, 2023).

The theoretical basis for this change of criteria was first formulated in the “Adaptation Level Theory” by Helson (1948). He described a frame of reference for the sensory-perceptive domain with which stimuli, like brightness of light and weights are evaluated. Helson (1948) noted that exposure to a certain stimulus intensity leads to habituation and thereby shifts the frame of reference. Heim et al. (2020b) transferred this principle to the linguistic domain, i.e., to the words for quantities instead of the quantities themselves. They demonstrated that the internal criteria for quantifiers which depend on our inner frame of reference can also be altered by habituation. In this study participants were shown pictures with different quantities of blue and yellow circles combined with a sentence containing “few” or “many.” Participants then had to judge whether the sentence correctly describes the picture. By limiting the stimuli range, i.e., only showing smaller proportions of the target color, the criteria of quantifiers were successfully shifted. And even though only one quantifier was trained to be accepted at lower proportions the evaluation of the other untrained quantifier changed as well. This illustrates that a semantic shift affects the entire frame of reference and thus also changes the criteria of the other quantifier. A change of meaning was also successfully induced in several previous experiments involving feedback, i.e., explicit reinforcement instead of adaptation (Heim et al., 2015, 2016, 2020a). Again, quantifier semantics have been modified and adapted to different ranges of proportions. In this study we complement and extend these investigations by examining a new group of subjects, who are patients with aphasia.

### Quantifier processing and related cognitive-linguistic functions in people with aphasia

A stroke can cause aphasia, i.e., damage to the language system. The symptoms of aphasia after stroke vary widely and can affect all aspects of language, from speech production and comprehension to reading, writing and any combination (Behrns et al., 2010; Fridriksson et al., 2018; Le and Lui, 2022). The localization, size and form of underlying lesions are correspondingly diverse, and the impairments and language fluency differ widely depending on lesion (Turken and Dronkers, 2011; Ardila et al., 2016; Døli et al., 2021). Since the left inferior frontal cortex, and in particular area 45 in Broca’s region, are involved in accessing and processing semantic representations of quantifiers (e.g., McMillan et al., 2005; Heim et al., 2012, 2016; Ash et al., 2016; for the role of the left insula located medially to Broca’s region, cf. Grodzinsky et al., 2020), damage to the left inferior frontal cortex impairs semantic evaluation (e.g., McMillan et al., 2006; Morgan et al., 2011). It can therefore be assumed that people with non-fluent aphasia after stroke, which is usually associated with damage to the left inferior frontal cortex (Benson, 1967; Bonilha and Fridriksson, 2009; Kasselimis et al., 2015; Yourganov et al., 2015; Biesbroek et al., 2016, 2021), exhibit pronounced difficulties in the tasks testing semantic flexibility in quantifier processing. The choice of participants, i.e., people with different types of post-stroke aphasia with different underlying lesions, could therefore provide interesting insights into the processing of quantifiers. In the next paragraph, we will give a short overview of the implications of different lesion sites for differential impairments of cognitive-linguistic functions.

Since quantifiers are words and must be assessed in the context of sentences, their processing is based on the linguistic system. This includes phonological, semantic, and syntactic processing as well as executive functions such as working memory (Mirman and Thye, 2018; Garraffa and Fyndanis, 2020; Lwi et al., 2021; Akkad et al., 2023). The experiments in this study involve the evaluation of sentences containing a quantifier, e.g., “Many of the circles are blue” associated with a picture containing a certain amount of blue and yellow circles. Successful completion of the task therefore requires the processes involved in sentence comprehension (i.e., phonetic and phonological analysis, global and local syntactic structure building, semantic expectation and comparison with incoming information, processes of integration and repair, and also working memory; for a complex neurocognitive model of sentence comprehension, cf., Friederici, 2002, 2017; passim). In addition, the linguistic processing of negative quantifiers involves implicit negation (cf. Grodzinsky et al., 2021; for the comparison to non-linguistic magnitude processing see Deschamps et al., 2015). Moreover, this particular paradigm also requires aspects of visual cognition and attention needed for the Estimation of the magnitude of the set of circles of the target color as well as its Comparison with the complement set, as well as access to numerical knowledge along the mental number line (cf. Heim et al., 2012). Finally, for the adaptation process, semantic flexibility is required (please note that the exact nature of this flexibility and its relation to other aspects of, e.g., executive functions is still under investigation; for a discussion of the relevance of individual semantic features vs. comprehensive categories cf. Thompson-Schill, 2003). All these functions may be impaired in post-stroke aphasia, depending on lesion location (Mirman and Thye, 2018; for a comparison of anterior vs. posterior lesions, cf. Stockert and Saur, 2017; and Stockert et al., 2020).

Since the experiments consist of hundreds of identically structured trials (sentence-picture-pairs), each trial has similar requirements for phonological and syntactical processing. The variables that change are quantifier (“few”/ “many”) and proportions of circles of the target color set. This variation places greater demands on semantic processing in terms of a truth value judgment linking the sentence to the visual display of colored circles. In terms of lesion site, the left IFG is of particular interest, since it has been identified as crucial area not only in semantic quantifier processing (McMillan et al., 2005; Heim et al., 2012, 2016; Ash et al., 2016; for the role of the left insula located medially to Broca’s region, cf. Grodzinsky et al., 2020) but also syntactic and phonological processing (for reviews, see Hagoort, 2005; Hagoort and Indefrey, 2014). In contrast, the posterior part of the perisylvian language network [i.e., Wernicke’s region and the temporo-parietal junction area; for the (im)precision of the concept of “Wernicke’s area” in the literature see Mesulam et al., 2015] was shown to be primarily involved in the Estimation and Comparison phases (Heim et al., 2012). Since both the left *and* the right hemisphere contribute, a posterior lesion is probably less severe for quantifier processing than a left frontal lesion for which the right homolog is less prepared to compensate (see, e.g., the review by Wilson et al., 2019). This, in turn, means we would expect greater deficits, i.e., poorer performance when a lesion affects this frontal area, which is more likely affected in non-fluent aphasia (Benson, 1967; Bonilha and Fridriksson, 2009; Kasselimis et al., 2015; Yourganov et al., 2015; Biesbroek et al., 2016, 2021). Finally, in Broca’s region, several functionally distinct modules for semantic, syntactic, morphological, and phonological processing are located very closely to each other (e.g., Hagoort, 2005; Hagoort and Indefrey, 2014; Friederici, 2017), so frontal lesions are usually associated with both lexical and morphosyntactic deficits (which characterize Broca’s aphasia). To what extent the processing of quantifiers, and in particular of negative quantifiers, involves or even critically relies on (implicit) syntactic operations is a matter of discussion (for a recent review and discussion, cf., Brasoveanu and Dotlacil, 2019). The non-linearity observed in behavioral data when parametrically increasing the number of negations in quantifier-containing sentences (Grodzinsky et al., 2021) might at least speak against linear-incremental syntactic complexity.

The questions arise whether people with fluent and non-fluent aphasia are capable of performing the task in general and whether they show a significant semantic flexibility effect, i.e., a systematic shift of the internal criterion, as was observed in the previous studies (Heim et al., 2015, 2016, 2020a,b). Because of the considerations above we furthermore distinguish between patients with fluent aphasia (PWFA) and patients with non-fluent aphasia (PWNFA). The hypothesis based on the thoughts above would be that PWNFA demonstrate poorer results evaluating quantifiers and may also lack semantic flexibility whereas PWFA may perform comparatively better.

In two experiments we examined semantic quantifier flexibility in people of both groups using the two quantifiers “many” and “few” with different manipulations of quantifier semantics, i.e., (implicit) adaptation (as in Heim et al., 2020b) and reinforcement learning (as in Heim et al., 2015, 2016, 2020a).

## Methods

The experiments were assigned to the participants in a counterbalanced order, i.e., some participants started with Experiment 1, some with Experiment 2. They were performed at least three days apart from each other usually at intervals of one week. Both experiments were conducted in German and performed on a computer using the Presentation software, version 23.0. Audio was played over a JBL Box to ensure sufficient sound volume.

Both experiments were approved by the ethics committee of RWTH Aachen University (EK 391/21). Informed written consent was obtained from all participants. Capability to consent was verified by the supervising senior physician.

### Participants

Twenty-one persons diagnosed with post-stroke aphasia participated in this study. All were patients at the Uniklinik RWTH Aachen (University Hospital Aachen). The study included five women and 16 men, ranged 26–74 years (average: 56 years). They had received an average of 16 years of education (range 11–18 years). Among all patients were 15 who reported to be right-handed, four left-handed and two ambidextrous. Regarding syndromes, four PWA showed global aphasia, one was diagnosed with Wernicke’s aphasia, three with Broca’s aphasia, six with amnestic aphasia, and three with residual aphasia. One patient was diagnosed with fluid aphasia with word finding and word processing disorders. In three cases, the aphasia was unclassifiable. Aphasia was post-acute in 11 cases and chronic in 10. In two persons aphasia was caused by atypical left intracerebral hemorrhage while ischemia was reported in two patients. In two others there was left sinus vein thrombosis. Left cerebral infarction was described in 13 patients without precise etiologic differentiation between hemorrhage and ischemia. In two cases, dissection with consecutive left-sided infarction was diagnosed. Because all patients were no longer in the acute phase and had therefore not been diagnosed during the same stay, no imaging data was available and data on etiology had to be based on written reports, which differed largely in their level of detail. Unfortunately, most of the patients had no structural images of their brains taken previously (CT, MRI). For research purposes, the ethics approval did not cover additional brain scans. Moreover, the number of eligible participants would have been reduced significantly due to potential contraindications. For these reasons, the present study does not feature any anatomical images of the brain lesions but solely refers to the syndrome and observable state of fluency of the people with aphasia (PWA). Table 1 provides the clinical details of the participants.

**Table 1:** Overview of the persons initially included in the study [clinical syndrome and severity and neuropsychological tests of working memory (Corsi Block Tapping Test: percentile; Non-Verbal Learning Test (NVLT): Percentile) and executive function (Go/No-Go Errors: Percentile)].

Syndrome (AAT)	Type of aphasia	Severity (overall)	Age group	CORSI	NVLT	Go/No-Go	Participant
Amnestic	Fluent	Mild/moderate	60–64	37	29	62	Y
Amnestic	Fluent	Mild/moderate	70–74	37	50	18	Y
Amnestic	Fluent	Moderate	50–54	85	43	58	Y
Amnestic	Fluent	n.a.	25–29	76	72	58	Y
Amnestic	Fluent	Moderate	50–54	64	2	27	Y
Amnestic/residual	Fluent	n.a.	60–64	37	60	63	Y
Broca	Non-fluent	Moderate	55–59	65	31	62	Y
Broca	Non-fluent	Moderate	55–59	37	26	34	Y
Global	Non-fluent	Moderate	55–59	43	25	63	Y
Non-classifiable	Fluent	n.a.	50–54	3	31	63	Y
Non-classifiable	Fluent	n.a.	50–54	86	51	58	Y
Non-classifiable	Non-fluent	Moderate	65–69	86	68	34	Y
Non-classifiable	Non-fluent	Moderate	45–49	86	57	67	Y
Residual	Fluent	n.a.	65–69	64	25	34	Y
Residual	Fluent	n.a.	55–59	37	5	34	Y
Residual	Fluent	n.a.	55–59			62	Y
*Broca*	*Non-fluent*	*Moderate*	*60–64*	*4*	*43*	*18*	*N*
*Global*	*Non-fluent*	*Mild/moderate*	*60–64*	*39*	*68*	*58*	*N*
*Global*	*Non-fluent*	*Severe*	*45–49*	*64*	*22*	*63*	*N*
*Global*	*Non-fluent*	*Severe*	*55–59*	*37*	*2*	*63*	*N*
*Wernicke*	*Fluent*	*Moderate/severe*	*70–74*	*65*	*4*	*34*	*N*

Note that percentiles below 16 indicate performance below average. n.a., not assigned; N, no (dropped out after initial trial run); Y, yes (participated). Italicized values and terms: Values of patients with aphasia who did not qualify for participation in the practice rounds = non-participants.

Criteria for participation included legal age (18 years or older), German language skills at native speaker level, time since stroke at least 6 weeks, no other independent language impairing neurological diseases (dementia or severe microangiopathy etc.) and the ability to give informed consent. The ability to give informed consent was assessed by the supervising senior physician (author JP).

**Figure 1:**
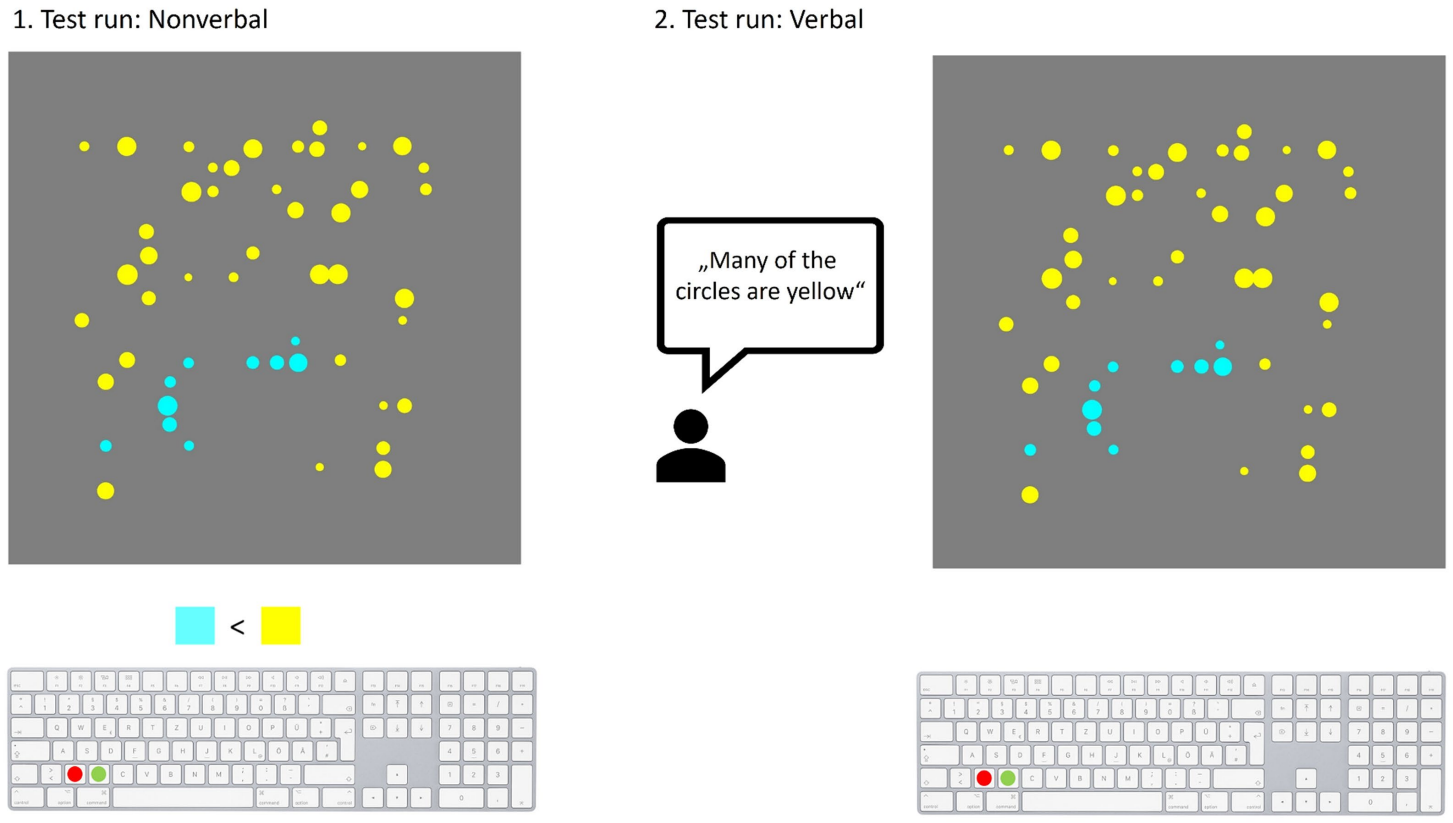
Schematic illustration of practice items in both test runs. First test run with a nonverbal version of the task **(Left)**. PWA were presented with a printed version of a picture with blue and yellow circles with a mathematical expression underneath indicating that one color outweighs the other. The patients were instructed to evaluate whether the expression matched the picture or not. In preparation for the computer version of the experiment a keyboard was also depicted. In the second test run **(Right)** a sentence containing a quantifier was spoken by the examiner. Following this, a picture was presented in printed version. The patient then had to decide whether the sentence adequately described the picture.

**Figure 2:**
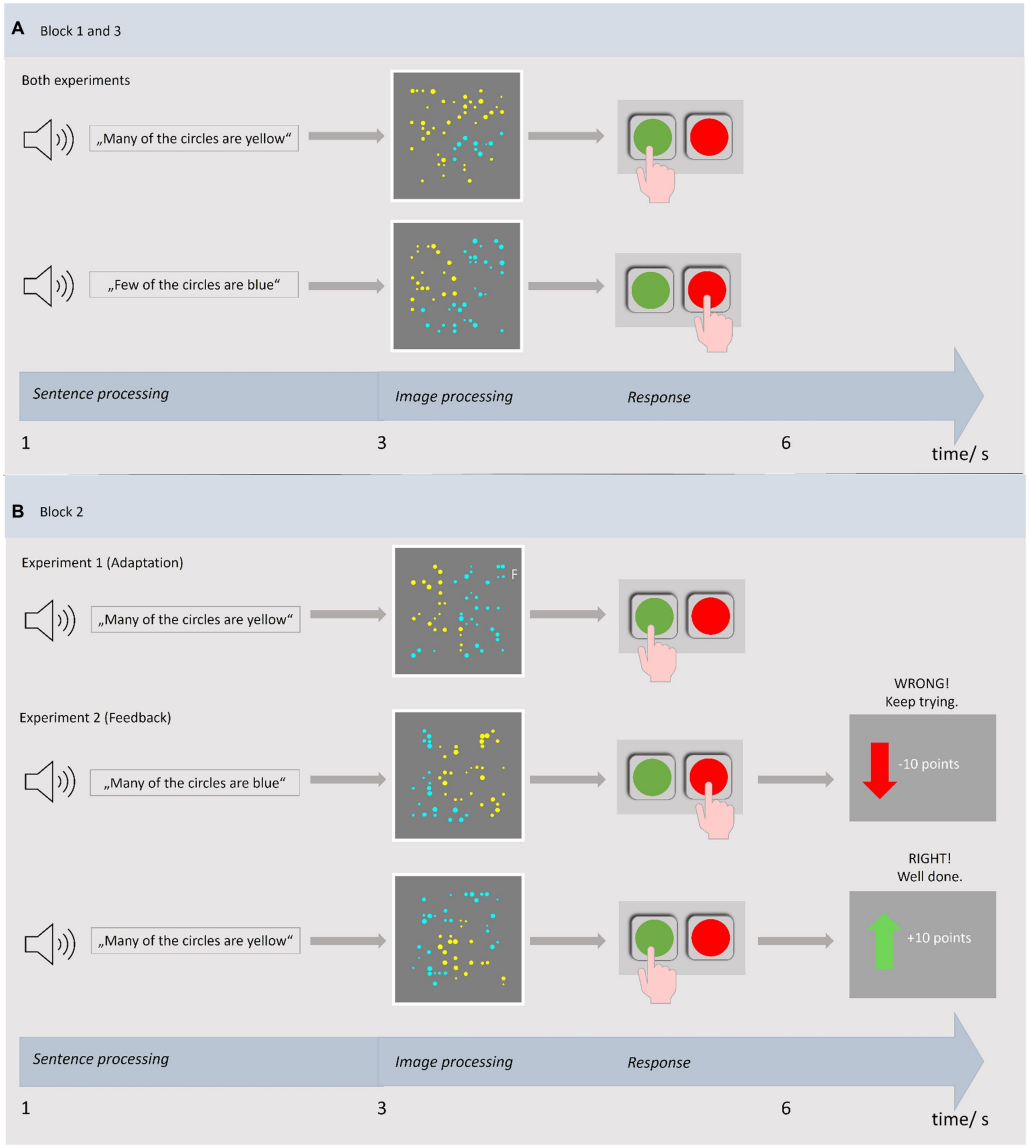
Exemplary trials of blocks 1 and 3 **(A)** and of block 2 **(B)** in both experiments based on Heim et al. (2012, 2015).

All patients were hospitalized for intensified aphasia therapy at the time of the study and participated during their free hours. Their therapy was not affected at any time by the participation in the study. To assess their general ability to perform the tasks each patient was required to qualify for participation in the computer experiments by judging practice items in two rounds (please see below). Patients qualified for participation if they correctly evaluated at least five of eight practice items in both rounds (Figure 1). All those who rated less correctly did not further participate in the computer experiments and are hereafter referred to as non-participants. Of 21 patients, 16 patients qualified for participation in the experiment while five did not qualify. The final sample therefore consists of 16 people with aphasia. Out of these, one person performed below average on the Corsi Block Tapping Test (visual working memory) and two others on the Non-Verbal Learning Test, but no participant consistently performed below average on both tests. Moreover, the Go/No-Go test of the TAP battery for attention revealed no overall executive deficit for any of the participants (Table 1). One participant was only able to perform the first block in both experiments due to mental exhaustion. These data were only included in calculations on Block 1, which means that the data from 16 participants were included in the calculations for Block 1 and the data from 15 participants in the calculations for Blocks 1 and 3. Figure 2 gives an overview of the paradigms.

## Experiment 1

The aim of Experiment 1 was to determine the internal criteria for the evaluation of “many” and “few” in PWA and whether the criterion for “many” could be shifted specifically toward smaller proportions by adaptation. Furthermore, we tested if this shift also extended to the untrained quantifier “few.”

### Procedure

Prior to the computer experiments all participants completed two test runs. For this purpose, selected stimuli (pictures with blue and yellow circles) from the experiments were shown in printed form and served as practice items. Each test run included eight items that had to be evaluated. See below for details on stimuli and procedure in the computer experiments. In the first practice round pictures were paired with a non-verbal expression including a comparison sign and in the second round with a quantifier containing sentence spoken by the examiner (Figure 1). This way, non-verbal quantification skills could be assessed first since linguistic interpretation was not yet required. If a patient was unable to evaluate the images, this step-by-step procedure allowed to determine whether the failure was caused by a lack of linguistic ability. Only participants who evaluated more than half of the items (at least five) correctly in both the non-verbal and verbal round and thus achieved more than guess probability qualified for participation in the computer experiments. Patients who evaluated fewer items correctly did not participate further and are referred to as non-participants. Both practice rounds as well as the computer experiments contained a truth value judgment task (cf. Figure 2) adapted from Heim et al. (2012). It was previously used in an adaptation study with neurotypical participants (Heim et al., 2020b). The task required the evaluation of sentence-picture pairs. Every sentence contained the quantifier “many” or “few” (e.g., “Many of the circles are blue”) and was presented auditorily in combination with a black screen. Each sentence was followed by a visual stimulus, i.e., a picture with different proportions of blue and yellow circles. For exact details on time sequences see Heim et al. (2020b). The stimulus consisted of a total of 50 monochrome circles of different diameters on a gray background. The proportions of the two colors varied and ranged from 20/30/40/50/60/70/80%. In the stimuli shown as practice items, one color always outweighed the other (ratio 20/80; 30/70; 40/60). I.e., no 50/50 picture was shown in which case no correct or incorrect answer would have been possible. For each proportion existed six different stimuli pictures to avoid facilitated recognition by repeating the exact same image. These stimuli versions were presented in a pseudo-randomized order. Participants decided whether the previously heard sentence adequately described the depicted distribution of colored circles by pressing a response button on the computer keyboard (YES button marked with a round green sticker, NO with a red one). As in the study of Heim et al. (2015) the position of the response button was alternated between patients but remained the same for each patient across experiments. This was intended to minimize possible influence by effects that facilitate answering with a particular response side [for discussion of the Spatial-Linguistic Association of Response Codes (SLARC) effect see Abbondanza et al. (2021)]. In addition, all participants were instructed to respond with the left hand to avoid, as far as possible, bias in the results due to motor impairments/paresis resulting from stroke. The experiment consisted of three blocks (Heim et al., 2020b) with a total of 392 trials and took approximately 1 h to complete. Participants started the computer experiment with six practice trials to become familiar with the task in the digital version. The subsequent baseline block including 112 trials recorded the patient’s initial judgment behavior allowing insights on the internal criteria for quantifier evaluation. This was followed by a training block with 168 trials. The task remained the same, but the stimuli range of the target color was limited to lower quantities (20–50%) and only the quantifier “many” was used. According to adaptation level theory (Helson, 1948) and previous findings (Heim et al., 2020b) habituation to the new stimulus range should cause a shift of the frame of reference, thereby shifting the internal criteria for “many” toward lower proportions. As a result, acceptance of lower proportions, e.g., 40% of the target color, as “many” is expected to increase. And if generalization to the untrained quantifier occurs, a decrease in acceptance of “few” would be anticipated. The third block (test block) was identical to the baseline block and recorded any change of quantifier semantics due to adaptation in the second block. Since both quantifiers were used again, differences in evaluation could be registered for the trained quantifier “many” as well as for the untrained quantifier “few.” Because the experiment was cognitively demanding, four 2-min breaks were scheduled to give time for rest.

During the experiment, acceptability judgments of quantifiers and reaction time (RT) were measured. Participants were instructed to respond correctly and as quickly as possible.

### Data analysis

For analysis purposes participants were divided into two groups according to their profile of spontaneous speech ratings in the Aachener Aphasie Test (Aachen Aphasia Test, AAT; Huber et al., 1983). It is the gold standard and most widely used instrument in German-speaking countries for the diagnosis of aphasia (Huber et al., 1983; Wacker et al., 2002). Following the rationale by Lange et al. (2012), patients with a score of at least three in syntax and four in articulation were classified as people with fluent aphasia (PWFA) and all patients with scores below that as people with non-fluent aphasia (PWNFA). The statistical analyses were conducted with IBM SPSS 27.

#### Reaction times

In order to test whether the polarity effect, i.e., longer reaction times for negative than positive quantifiers, was also present in PWA, a linear mixed model (LMM) with “subject” as random factor and GROUP (PWFA/PWNFA), BLOCK (Block 1/Block 3), and PROPORTION (20, 30, 40, 50, 60, 70, and 80) as fixed factors was conducted. Since the participants had not explicitly been instructed to perform the task as a speed task, and since the RT data in the previous studies did not contribute to the understanding of semantic flexibility in quantifier processing, all other main effects and interactions are of no primary interest in this analysis.

#### Acceptability

For the purpose of this study, only the acceptability judgments of the PWA for the critical proportion “40%” were relevant. The trials featuring other proportions of circles of the named color only had the function of filler trials in the context of the potential adaptation of the participants’ responses. For this reason, the analysis of the acceptability judgments included a LMM with “subject” as random factor and GROUP (PWFA/PWNFA) and BLOCK (Block 1/Block 3) as fixed factors. Only responses for the proportion “40%” of circles of the named color were analyzed. The focus of this study was on the question whether the acceptability for the trained quantifier (“many”) changes significantly from Block 1 to Block 3, and whether this change also impacts acceptability ratings for the untrained quantifier “few.” Next, the directed (one-tailed) pair-wise comparisons of “Block 1 vs. Block 3” were calculated at proportion “40%” separately for each group and each quantifier after using the split-file command in SPSS.

Non-responses, i.e., all trials in which a participant did not respond in the given time, are reported but were not included in the analysis. Although non-responses also carry a certain informational value it is possible that some or many are caused mainly by exhaustion of attention and concentration. Since the study was mostly concerned with the actual evaluation of quantifiers, i.e., their conscious acceptance and rejection non-responses were excluded to avoid bias due to exhaustion.

### Results: Experiment 1

#### Reaction times

**Figure 3:**
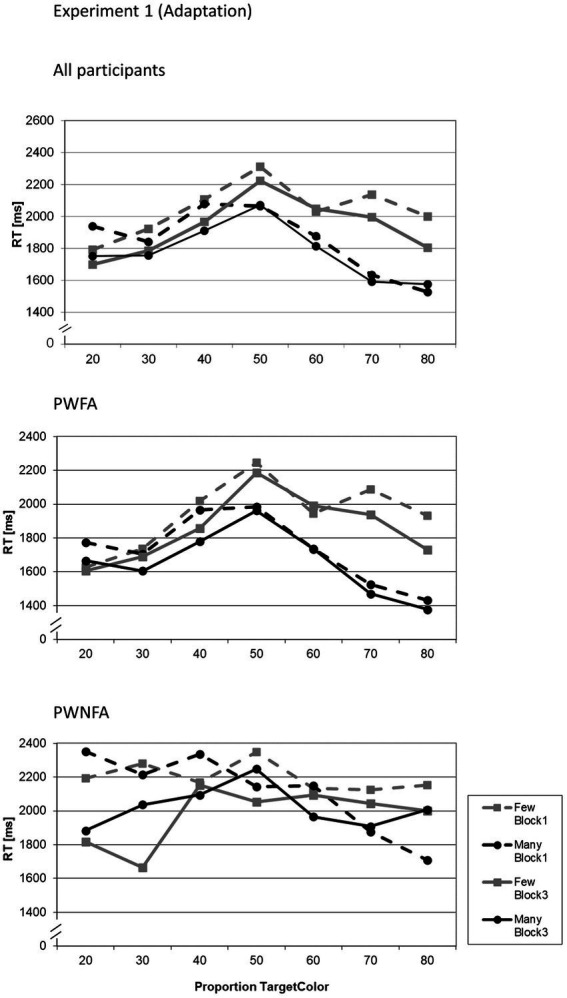
Average reaction times of all participants in Experiment 1 (adaptation) for both quantifiers (“many”, “few”) related to each proportion of circles in the target color (in %), divided in groups: all participants, PWFA and PWNFA. Reaction times are presented for blocks 1 and 3, before and after adaptation to visualize changes in the course.

The LMM yielded significant main effects for GROUP (*F*_1;3,156_ = 86.256; *p* < 0.001), QUANTIFIER (*F*_1;3,156_ = 14.169; *p* < 0.001), BLOCK (*F*_1;3,156_ = 15.729; *p* < 0.001), and PROPORTION (*F*_1;3,156_ = 9.509; *p* < 0.001). Out of the interaction terms, the following effects also reached significance: GROUP × QUANTIFIER (*F*_1;3,156_ = 9.278; *p* = 0.002), and QUANTIFIER × PROPORTION (*F*_1;3,156_ = 5.074; *p* < 0.001). All other effects were not significant. Table 2 reports the parameter estimates of the LMM. Figure 3 shows the RT as a function of BLOCK, QUANTIFIER and PROPORTION. With respect to the Polarity Effect in the RTs, PWFA had consistently higher RTs for “few” than for “many.” For PWNFA, the pattern was inconsistent. The QUANTIFIER × PROPORTION interaction was due to significant differences between the RTs for the two quantifiers at proportions 70 and 80% (both *p* < 0.001), while there were no differences at the other proportions (all *p* > 0.05 uncorrected). Figure 3 shows the reaction time data for the full sample and separately for PWFA and PWNFA.

**Table 2:** Parameter estimates of the LMM for RTs in Experiment 1.

Parameter	Estimate	SEM	df	*t*	*p*	95% CI	
						Lower	Upper
Constant Term	2008.033333	134.477797	3,156.000	14.932	0.000	1744.360573	2271.706094
GROUP	−630.791954	155.949466	3,156.000	−4.045	0.000	−936.564557	−325.019351
BLOCK	−300.133333	190.180325	3,156.000	−1.578	0.115	−673.022927	72.756261
QUANTIFIER	−8.613978	188.640378	3,156.000	−0.046	0.964	−378.484174	361.256217
[PROPORTION = 20]	−124.885185	195.391710	3,156.000	−0.639	0.523	−507.992824	258.222454
[PROPORTION = 30]	29.395238	193.546610	3,156.000	0.152	0.879	−350.094684	408.885160
[PROPORTION = 40]	85.735897	197.359450	3,156.000	0.434	0.664	−301.229922	472.701717
[PROPORTION = 50]	239.600000	190.180325	3,156.000	1.260	0.208	−133.289594	612.489594
[PROPORTION = 60]	−43.343678	191.812804	3,156.000	−0.226	0.821	−419.434100	332.746743
[PROPORTION = 70]	−100.783333	187.185172	3,156.000	−0.538	0.590	−467.800283	266.233616

Note that only the parameters for the main effects are reported here, since the sole focus is on the polarity effect. The full list of parameters can be found in Appendix A1.

#### Acceptability ratings

**Figure 4:**
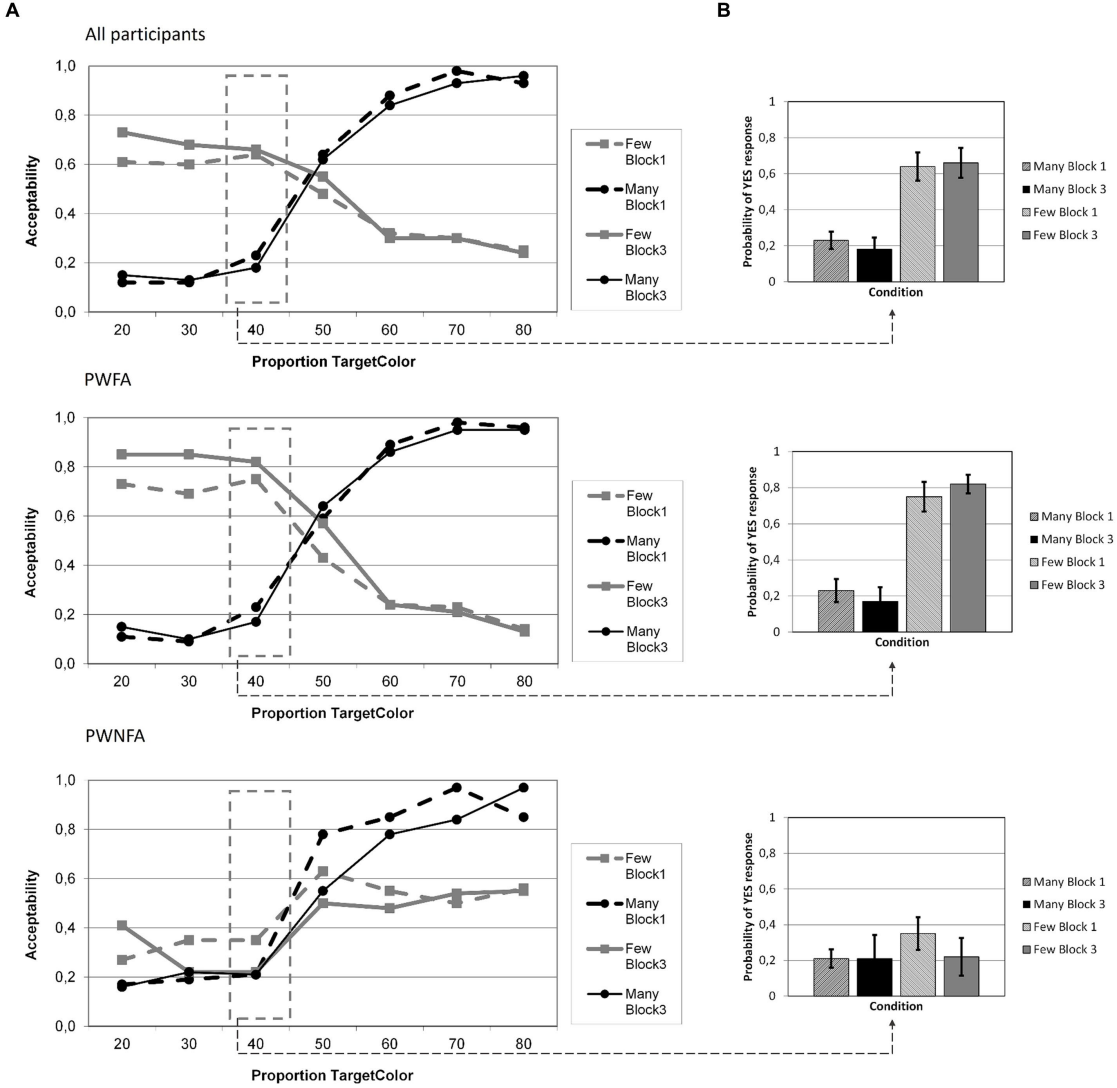
Average acceptability of quantifiers in Experiment 1 (adaptation), divided in groups: all participants, PWFA and PWNFA. **(A)** Illustration of acceptability of quantifiers (“many” = black lines, “few” = gray lines) at each proportion of circles in the target color (in %) in block 1 (dashed lines) and block 3 (solid lines). **(B)** Average acceptability judgments for the critical proportion of circles in the target color (40%), sorted by quantifier (“many” = black bars, “few” = gray bars) and block (block 1 = dashed bars, block 3 = solid bars).

The Linear Mixed Model for the acceptability ratings at proportion 40% yielded significant effects for GROUP (*F*_1;446_ = 27.252; *p* < 0.001) and QUANTIFIER (*F*_1;446_ = 59.884; *p* < 0.001) and a significant interaction GROUP × QUANTIFIER (*F*_1;446_ = 25.749; *p* < 0.001). The main effect for, and interactions with BLOCK failed to reach significance (BLOCK: *F*_1;446_ = 0.416; *p* = 0.519; BLOCK × QUANTIFIER: *F*_1;446_ = 0.001; *p* = 0.973; BLOCK × GROUP: *F*_1;446_ = 0.357; *p* = 0.551; BLOCK × QUANTIFIER × GROUP: *F*_1;446_ = 2.051; *p* = 0.153). The parameter estimates are reported in Table 3, the ratings per condition and block in Figure 4. Across the entire group of participants, there were 4.5% (271) non-responses.

**Table 3:** Parameter estimates for the LMM analysis for the acceptability ratings at proportion “40%” for Experiment 1.

Parameter	Estimate	SEM	df	*t*	*p*	95% CI	
						Lower	Upper
Constant term	0.192308	0.081081	446.000	2.372	0.018	0.032960	0.351656
GROUP	−0.031388	0.092405	446.000	−0.340	0.734	−0.212992	0.150216
BLOCK	−0.007123	0.113599	446.000	−0.063	0.950	−0.230378	0.216133
QUANTIFIER	0.057692	0.112599	446.000	0.512	0.609	−0.163599	0.278984
GROUP × BLOCK	0.076088	0.129746	446.000	0.586	0.558	−0.178902	0.331078
GROUP × QUANTIFIER	0.593153	0.129051	446.000	4.596	0.000	0.339529	0.846777
BLOCK × QUANTIFIER	0.127493	0.159185	446.000	0.801	0.424	−0.185353	0.440339
GROUP × BLOCK × QUANTIFIER	−0.261097	0.182332	446.000	−1.432	0.153	−0.619432	0.097239

Importantly, none of the planned directed linear contrasts for each group and quantifier reached (one-tailed) significance (PWFA, “many”: *p* = 0.127; PWFA, “few”: *p* = 0.155; PWNFA, “many”: *p* = 0.474; PWNFA, “few”: *p* = 0.172). Both groups showed significant differences for their rating of “few” (Block 1: *p* < 0.001; Block 3: *p* < 0.001) but not for “many” (Block 1: *p* = 0.624; Block 3: *p* = 0.734) at the critical proportion 40%.

For the sake of direct comparability with the previous studies using the same paradigms (Heim et al., 2015, 2016, 2020a,b), the classic ANOVA on aggregated data was also conducted and is reported in Appendix A2.

### Discussion: Experiment 1

The purpose of this experiment was to investigate the general processing of quantifiers in PWA and their ability to change the meaning of the quantifier “many” through adaptation. The results show a significant difference in the acceptance of the two quantifiers and the speed with which they were evaluated. Participants responded comparatively faster to “many” and accepted it more often than “few.” There was no semantic shift due to adaptation in any group. It has been suggested that the processing of negation (even if implicit in a negative quantifier) takes longer, because it is more costly, i.e., cognitively more demanding (Just and Carpenter, 1971; Deschamps et al., 2015; Agmon et al., 2021; Grodzinsky et al., 2021). That this effect is found in PWA as well as in neurotypical individuals might indicate similar processing patterns in the patient group.

However, with respect to general *accuracy* (reflected in the acceptability judgments), it appears that the processing difficulty for the negative quantifier “few” in comparison to the positive quantifier “many” was more pronounced in PWNFA than in PWFA, as indicated by the significant GROUP × QUANTIFIER interaction at proportion 40% (Table 3). The corresponding graphs illustrating the acceptability judgments of PWNFA (Figure 4) reflect that this subgroup had generally more difficulties processing the negative quantifier “few”: While the “many”-curves approximate the expected course, the “few”-curves are inverted (in comparison to those of the PWFA and the neurotypical participants in the earlier studies), here following roughly the course of the “many”-curves. I.e., “few” is even more often accepted when larger proportions are shown. This pattern would be consistent with the notion that the PWNFA processed “few” in the same way as “many,” i.e., using “many” as the default and failing to add the implicit negation (few = “not many”). The implications will be discussed later in more detail.

The adaptation manipulation in Block 2 failed to induce a change in the evaluation of “many” at the critical proportion 40%. No semantic shift occurred for any group regardless of speech fluency, neither for “many” nor for “few.” Consequently, the results of PWA differ substantially from those of neurotypical individuals in a previous adaptation study (Heim et al., 2020b) who showed a successful semantic shift which even included a generalization to the untrained quantifier. Adaptation processes have thus been proven to be effective in principle in neurotypical individuals. The question arises whether the absence of a semantic shift is due either to a fundamental lack of semantic flexibility in PWA or only to dysfunctional adaptation processes while semantic flexibility is principally preserved. If the latter would be true, other learning methods such as direct reinforcement may be able to induce a shift. To answer this, we conducted Experiment 2 which involves feedback instead of adaptation.

## Experiment 2: feedback paradigm

With this experiment, we further investigated the internal criteria for quantifier evaluation and semantic flexibility of PWA. The question here was whether feedback (as opposed to adaptation) can trigger a semantic shift and change the criterion for “many” in the direction of lower proportions.

### Methods: Experiment 2

The stimuli originate again from Heim et al. (2012). The feedback paradigm was applied previously in several studies (Heim et al., 2015, 2016, 2020a). Although very similar in its structure, this experiment differs in some important respects from the Adaptation experiment described above. The most striking difference is the method of manipulation to shift the semantics of quantifiers. Instead of limiting stimuli range targeting adaptation processes, the second block provides feedback thereby using explicit reinforcement learning techniques to change the inner criteria. Here, too, the evaluation of “many” should be changed. Each of the three blocks included 168 trials. After the initial baseline block (Block 1), feedback was displayed in the training block (Block 2) immediately following each participant response (cf. Figure 2). In case of positive feedback points were given (+10 points) combined with a green arrow pointing upward and affirmative words in German language (“Correct! Well done.”). Accordingly, negative feedback consisted of a loss of points (−10 points) with a red arrow pointing downward and a negative verbal statement (“Wrong. Keep trying”). As in Experiment 1, only the quantifier “many” was used in the second block. Positive feedback was granted, when a proportion of circles of 40% or higher was evaluated as “many,” i.e., when participants responded with YES to a stimulus with at least 40% of the target color. Negative feedback was shown when participants decided otherwise. Thus, participants were trained to accept “many” for any amount greater than or equal to 40%. According to previous studies (Heim et al., 2015, 2016, 2020a) this feedback would effectively cause a shift of internal criteria for the trained quantifier (“many”), and when generalization takes place for the untrained quantifier “few” as well. For details on time sequences and feedback see Heim et al. (2015).

### Data analysis: Experiment 2

For Experiment 2 we proceeded with the analysis in the same way as for Experiment 1.

### Results: Experiment 2

#### Reaction times

**Figure 5:**
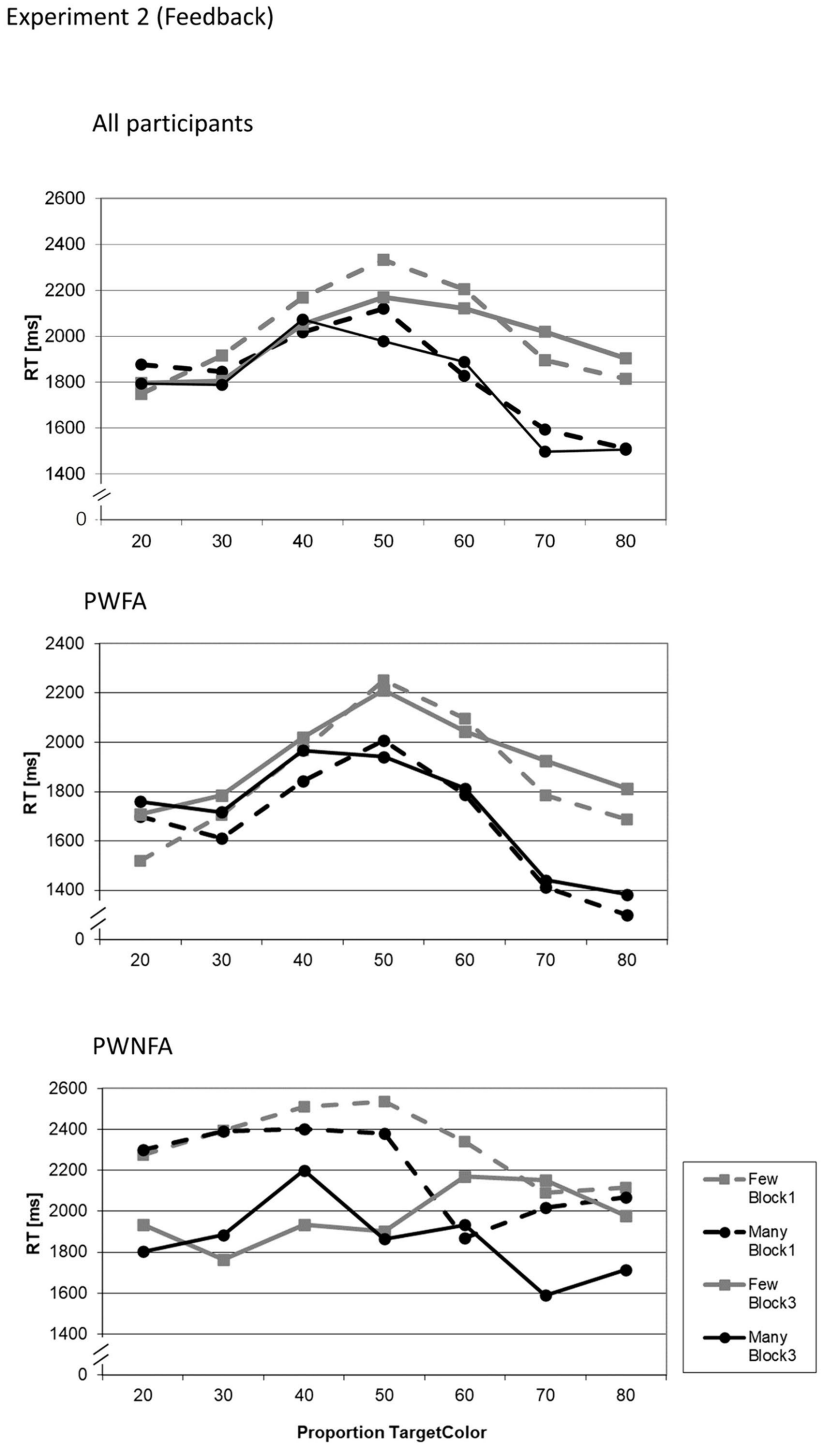
Average reaction times of all participants in Experiment 2 (feedback) for both quantifiers (“many”, “few”) related to each proportion of circles in the target color (in %), divided in groups: all participants, PWFA and PWNFA. Reaction times are presented for blocks 1 and 3, before and after feedback to visualize changes in the course.

The LMM yielded significant main effects for GROUP (*F*_1;4,715_ = 168.908; *p* < 0.001), QUANTIFIER (*F*_1;4,715_ = 49.832; *p* < 0.001), BLOCK (*F*_1;4,715_ = 39.391; *p* < 0.001), and PROPORTION (*F*_1;4,715_ = 23.415; *p* < 0.001). Out of the interaction terms, the following effects also reached significance: GROUP × BLOCK (*F*_1;4,715_ = 79.607; *p* < 0.001), GROUP × PROPORTION (*F*_1;4,715_ = 5.356; *p* < 0.001), BLOCK × PROPORTION (*F*_1;4,715_ = 2.935; *p* = 0.007), QUANTIFIER × PROPORTION (*F*_1;4,715_ = 7.588; *p* < 0.001), GROUP × BLOCK × PROPORTION (*F*_1;4,715_ = 2.945; *p* = 0.007), and BLOCK × QUANTIFIER × PROPORTION (*F*_1;4,715_ = 2.374; *p* = 0.027). All other effects were not significant. Table 4 reports the parameter estimates of the LMM. Figure 5 shows the RT as a function of BLOCK, QUANTIFIER and PROPORTION. With respect to the Polarity Effect, PWFA had consistently higher RTs for “few” (Block 1: 1858 ms; Block 3: 1928 ms) than for “many” (Block 1: 1665 ms; Block 3: 1716 ms). For PWNFA, the same pattern showed somewhat less pronounced (“few”: Block 1: 2321 ms; Block 3: 1977 ms; “many”: Block 1: 2,200 ms; Block 3: 1,849 ms).

**Table 4:** Parameter estimates of the LMM for RTs in Experiment 2.

Parameter	Estimate	SEM	df	*t*	*p*	95% CI	
						Lower	Upper
Constant term	1714.166667	103.240427	4715.000	16.604	<0.001	1511.767192	1916.566141
GROUP	−331.575758	118.532501	4715.000	−2.797	0.005	−563.954844	−99.196671
BLOCK	353.023810	146.004012	4715.000	2.418	0.016	66.787727	639.259892
QUANTIFIER	264.961538	148.785290	4715.000	1.781	0.075	−26.727150	556.650227
[PROPORTION = 20]	88.190476	146.004012	4715.000	0.604	0.546	−198.045606	374.426558
[PROPORTION = 30]	169.883333	147.817796	4715.000	1.149	0.251	−119.908613	459.675280
[PROPORTION = 40]	486.083333	151.965793	4715.000	3.199	0.001	188.159373	784.007294
[PROPORTION = 50]	151.345528	146.891582	4715.000	1.030	0.303	−136.630607	439.321664
[PROPORTION = 60]	219.443089	146.891582	4715.000	1.494	0.135	−68.533046	507.419225
[PROPORTION = 70]	−124.532520	146.891582	4715.000	−0.848	0.397	−412.508656	163.443615

Note that only the parameters for the main effects are reported here, since the sole focus is on the polarity effect. The full list of parameters can be found in Appendix B1.

#### Acceptability ratings

**Figure 6:**
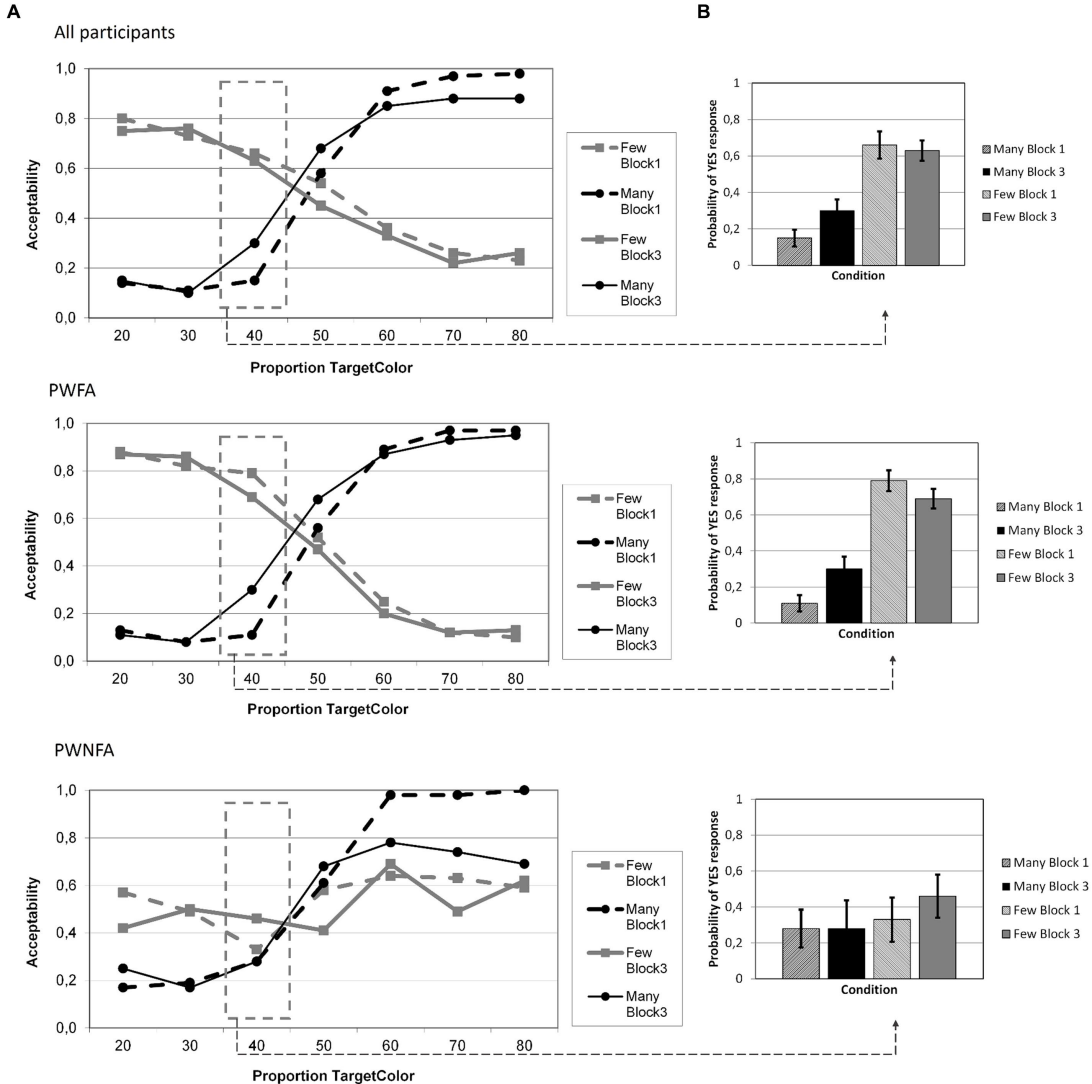
Average acceptability of quantifiers in Experiment 2 (feedback), divided in groups: all participants, PWFA and PWNFA. **(A)** Illustration of acceptability of quantifiers (“many” = black lines, “few” = gray lines) at each proportion of circles in the target color (in %) in block 1 (dashed lines) and block 3 (solid lines). **(B)** Average acceptability judgments for the critical proportion of circles in the target color (40%), sorted by quantifier (“many” = black bars, “few” = gray bars) and block (block 1 = dashed bars, block 3 = solid bars).

The Linear Mixed Model for the acceptability ratings at proportion 40% yielded significant effects for GROUP (*F*_1;665_ = 6.013; *p* = 0.014) and QUANTIFIER (*F*_1;665_ = 76,403; *p* < 0.001) and a significant interaction of GROUP × QUANTIFIER (*F*_1;665_ = 21.947; *p* < 0.001). The main effect for, and interactions with, BLOCK failed to reach significance (BLOCK: *F*_1;665_ = 2.993; *p* = 0.084; BLOCK × QUANTIFIER: *F*_1;665_ = 3.104; *p* = 0.079; BLOCK × GROUP: *F*_1;665_ = 0.261; *p* = 0.610; BLOCK × QUANTIFIER × GROUP: *F*_1;665_ = 3.624; *p* = 0.057). The parameter estimates are reported in Table 5, the data per quantifier and block in Figure 6. Across the entire group of participants, there were 4.7% (362) non-responses.

**Table 5:** Parameter estimates for the LMM analysis for the acceptability ratings at proportion “40%” for Experiment 2.

Parameter	Estimate	SEM	df	*t*	*p*	95% CI	
						Lower	Upper
Constant term	0.333333	0.071883	665.000	4.637	0.000	0.192188	0.474479
GROUP	−0.033333	0.081229	665.000	−0.410	0.682	−0.192829	0.126163
BLOCK	−0.083333	0.099084	665.000	−0.841	0.401	−0.277889	0.111223
QUANTIFIER	0.166667	0.099084	665.000	1.682	0.093	−0.027889	0.361223
GROUP × BLOCK	−0.110606	0.112507	665.000	−0.983	0.326	−0.331518	0.110306
GROUP × QUANTIFIER	0.220833	0.112703	665.000	1.959	0.050	−0.000463	0.442130
BLOCK × QUANTIFIER	−0.011261	0.139627	665.000	−0.081	0.936	−0.285425	0.262902
GROUP × BLOCK × QUANTIFIER	0.302316	0.158809	665.000	1.904	0.057	−0.009511	0.614143

Regarding the planned linear contrasts for each group and quantifier at proportion “40%,” (one-tailed) significance was observed for PWFA (“many”: *p* < 0.001; “few”: *p* = 0.039; these uncorrected *p*-values survive the Bonferroni–Holm correction within the group of PWFA) but not for PWNFA (“many”: *p* = 0.216; “few”: *p* = 0.206).

### Discussion: Experiment 2

The findings from Experiment 2 complement and extend the considerations from Experiment 1. Analyses of acceptability ratings and reaction times corroborated a significant difference between quantifier evaluation and replicated the presence of a polarity effect. Again, this effect seems more pronounced in PWNFA than in PWFA. Moreover, in contrast to the adaptation procedure of Experiment 1, feedback in Experiment 2 successfully elicited a semantic shift in a subgroup of the participants (cf. Figure 6): PWFA succeeded in adapting their inner criteria for the trained quantifier through feedback and additionally transferred this shift to the untrained quantifier, even though this effect was weaker and only present when not correcting the p-value for the number of planned linear contrasts (i.e., 2). PWNFA meanwhile showed no shift or transfer, as in Experiment 1. In terms of semantic shift, Experiment 2 provided different results than Experiment 1, thereby answering the previously posed question regarding the presence of semantic flexibility in PWA. While the entire group on average and PWNFA failed again to shift the criteria for quantifier evaluation, PWFA succeeded this time, in contrast to Experiment 1. Additionally, the shift not only affected the trained quantifier “many” but was even transferred to the untrained quantifier “few,” which had not been presented during the training block. This means that at least in PWFA, explicit reinforcement as opposed to adaptation can induce a shift, just as it does in neurotypical individuals (Heim et al., 2015, 2016). In contrast to the latter, however, this shift does not work by adaptation in patients with aphasia (Experiment 1).

As pointed out in a previous study (Heim et al., 2015), generalization implies a deeper learning level which allows to abstract and transfer semantic change of one quantifier to its polar opposite. Heim et al. (2015) argued that when acceptance of “many” at 40% is increased, it logically follows that the acceptance of “few” cannot remain the same but must decrease. Otherwise, two (quasi-polar) opposite quantifiers that semantically (at least partly, if processed as majority quantifiers) exclude each other would describe the same quantity, i.e., carry the same semantic information (Oaksford et al., 2002; Heim et al., 2015; Pezzelle et al., 2018). The change of meaning is particularly noteworthy because the exact same group of patients managed a shift when explicit reinforcement is used but not through adaptation processes alone. It follows that PWFA must in principle be semantically flexible because otherwise no shift could have occurred in Experiment 2. PWNFA, meanwhile, seem to lack this attribute as they did not show a shift in any experiment. The comparable learning success of PWFA and neurotypical individuals is also remarkable because it underlines the large differences in the performance spectrum of PWA regarding quantifier processing. Some PWA did not even qualify to participate, and some performed nearly as well as neurotypical participants.

### Further analysis: investigation of influencing factors regarding non-participants

**Figure 7:**
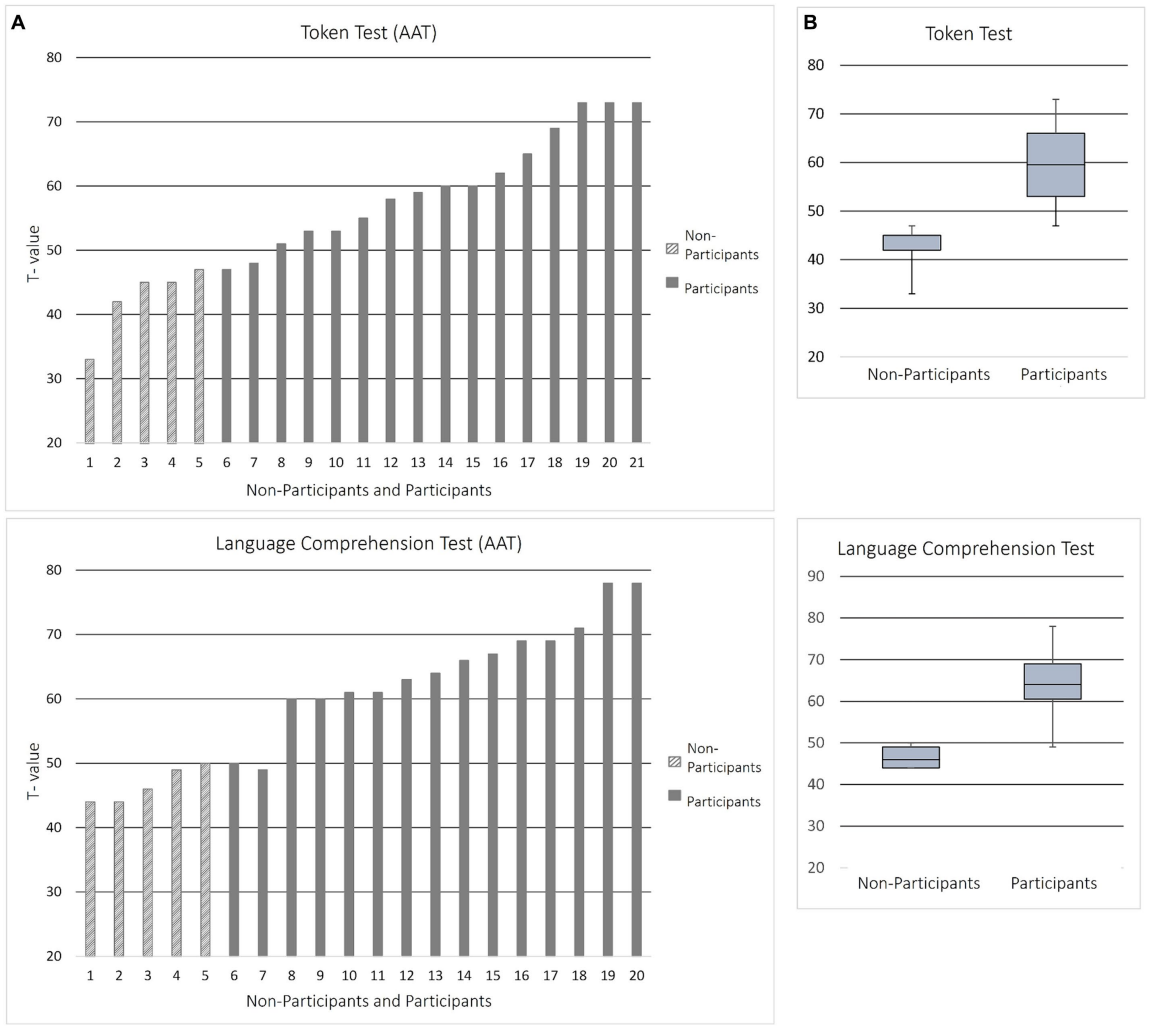
Performance of participants and non-participants in the token test and language comprehension test of the AAT in comparison. **(A)** Demonstration of the *T*-value achieved by each patient in both tests, plotted separately for non-participants (dashed columns) and participants (solid columns). **(B)** Average *T*-values achieved by non-participants and participants in both tests. With regard to the language comprehension test, in one case no test value was available, which is why only 20 instead of 21 test subjects are listed on the *x*-axis. The data are reported in ascending order of values. The participant numbers in panels **(A,B)** only indicate order of values and do not identify individual participants.

Before proceeding with the general discussion of the two experiments the cross-experiment tests and their results are reported. Not all PWA qualified in the two runs of practice trials to participate in the computer experiments. Five out of 21 PWA rated less than half of the practice items correctly, demonstrating insufficient ability to perform the task. Since this represents a distinctive difference to studies with neurotypical participants, we wanted to investigate the reasons for this in more detail. Therefore, we examined the non-participant group in comparison to participants. A Fisher test used to detect an association with fluency yielded a non-significant result (one tailed, *p* = 0.080) and thus no statistical connection. However, independent-samples *t*-tests comparing the performance of participants and non-participants in the AAT showed significant differences in the Token Test [*t*(19) = 4.200; *p* < 0.001] and the test of Language Comprehension [*t*(18) = 4.614; *p* < 0.001]. Participants performed noticeably better than non-participants (cf. Figure 7).

## General discussion

This study investigated quantifier processing and semantic flexibility in PWA. Results of both experiments showed varying degrees of limitations concerning quantifier processing in PWA. While 16 PWA were sufficiently capable of processing quantifiers to participate, five were too severely impaired. Among participants, performances differed significantly. PWNFA showed no semantic flexibility, whereas PWFA demonstrated a semantic shift when feedback was used. The adaptation paradigm failed to evoke a shift in the total sample and in either sub-group. However, in accordance with findings of neurotypical participants in the literature, a Polarity effect was observed, indicating higher processing costs for the negative quantifiers. We will now discuss the implications of these findings for quantifier processing in PWA.

### Semantic flexibility in quantifier processing in PWA

The central question of the present study was: Can the internal criterion for “many” be changed in PWA like in neurotypical individuals, i.e., do PWA show semantic flexibility? And if so, to which degree – does this semantic flexibility also extend, and thus generalize, to the polar opposite (here: “few”)? The statistical analysis yielded that no shift regardless of group (all, PWFA, PWNFA) occurred in Experiment 1. The curves illustrate this (cf. Figure 4): acceptability of neither “many” nor “few” at 40% shifted markedly in any direction. A similar result was found in Experiment 2, no semantic shift occurred in the group of PWA on average nor in the subgroup of PWNFA.

Importantly, however, PWFA did successfully change their criterion for “many.” They even generalized, i.e., transferred the change of internal criteria from the trained quantifier “many” to the untrained “few.” It follows that PWFA do not only show semantic flexibility but are able to transfer the learning success. As in neurotypical people, when the semantics of a quantifier is changed, it affects the entire quantifier scope as well, thus changing the criterion for the untrained quantifier (Heim et al., 2015). In contrast, PWNFA lack these abilities. The question thus emerges what role fluency of aphasia plays for the presence of semantic flexibility. And could it be that fluency of aphasia is not only related to the aspect of semantic flexibility, but also to quantifier processing in general?

### The relationship of fluency of aphasia and semantic flexibility

Fluency in aphasia is of particular importance in this study as it distinguishes two groups with very different results. The literature suggests that this could be related to a partial overlap between the neural basis of quantifier processing and semantic evaluation. The left IFG seems to be an integral area for both functions, fluency of aphasia (for a review see Mirman and Thye, 2018) and also semantic evaluation (Heim et al., 2012; McMillan et al., 2013; Wei et al., 2014) and re-evaluation of quantifiers (Heim et al., 2016, 2020a). Moreover, Broca’s region in the left IFG has been implicated in various types of semantic processing in the clinical and neuroimaging literature for 30 years. Such studies investigated, among others, access to categorial semantics (e.g., Martin et al., 1996; Vandenberghe et al., 1996), degrees of semantic control (e.g., Thompson-Schill et al., 1997), lexical access and retrieval (Damasio et al., 1996; Thompson-Schill et al., 1999; Amunts et al., 2004; Heim et al., 2009a,b; Hartwigsen, 2016), or semantic evaluation of sentences (Hagoort and van Berkum, 2007; for reviews cf. Binder et al., 2009; Ralph et al., 2017). While these studies seem to make a distinction between semantic representation (left temporal) and controlled access to, retrieval or evaluation of these representations (left inferior frontal), the underlying concepts of “semantics” and of “representation” or “processing” (etc.) vary substantially. What one can draw from the wealth of studies is the consistency of left inferior frontal involvement across a multitude of paradigms, which explicitly involves verbal fluency, and that damage to the left IFG may impair word retrieval (e.g., Thompson-Schill et al., 1998).

In the context of the findings of the present study, one might thus suspect that if a patient suffers from an impairment of speech fluency it could be possibly due to a lesion in this frontal region. Therefore, it would be conceivable that quantifier processing might be impaired as well because it is based on the same neural area. Conversely, one would suspect that if speech is fluent, there is more likely no frontal lesion (Benson, 1967; Pendleton et al., 1982; Cipolotti et al., 2021), and thus quantifier processing might also be less impaired. However, disorders of speech fluency can also be caused by lesions in other localizations than the left IFG, e.g., insula, precentral areas (Wilson et al., 2010; Fridriksson et al., 2013), anterior temporal lobe (Schwartz et al., 2009; Walker et al., 2011; Chen et al., 2019; Mirman et al., 2019) and inferior parietal regions (Rogalsky et al., 2015; Mirman et al., 2019) as well as white matter regions like the uncinate fasciculus, the anterior segment of the left arcuate fasciculus and the aslant tract (Catani et al., 2013; Fridriksson et al., 2013; Basilakos et al., 2014; Yourganov et al., 2015). Therefore, frontal areas relevant for quantifiers may well be intact even if speech fluency is impaired.

The present study contributes to the literature that fluency in aphasia seems to be a relevant factor. Since this is not a neuroimaging study, and exact information about lesion location and size was not available from the clinical records, a logical next step would be to transfer the feedback paradigm used in Experiment 2 into the scanner in order to gain further insights into the alterations of functional neuroanatomy of quantifier processing in aphasia.

Such a functional neuroimaging study would also provide the chance to investigate the nature of the difficulty of the PWNFA to process negative quantifiers: Is this an issue of the semantic representation of quantifiers *per se*, or rather, of their semantic evaluation? As pointed out above, in the domain of semantic processing, many different aspects have been attributed to different parts of Broca’s region in the IFG. Most of these aspects are related to “processing,” i.e., controlled, deliberate or increasingly difficult retrieval of, or access to, semantic information which seems rather “stored,” or represented, in (among others) the left temporal lobe, predominantly its inferior part, with the temporal pole as a potential hub (e.g., Ralph et al., 2017). If one supposes that a non-fluent variant of aphasia is likely caused by a lesion to Broca’s region and its surroundings (but see the difficulties of such reasoning elaborated above), this would imply difficulties in quantifier “processing,” presumably their semantic evaluation, rather than their representation. Several studies in the literature support this view. (1) In their neuroimaging study, Heim et al. (2012) adapted the triple code model by Dehaene et al. (2005) to quantifier processing in a truth value judgment task like the one used in the present study. The first two stages, estimation (of the size/magnitude of the set of circles in the given color) and comparison (of that set to the complement set), were supported by a large, mostly fronto-parietal, network in the left and also in the right hemisphere. In contrast, the Polarity effect, taken as a proxy for the semantic evaluation (i.e., the third stage), was very focal in area 45 of Broca’s region. This location of the Polarity effect was later replicated by Agmon et al. (2021). The findings are in line with the earlier observation by McMillan et al. (2005) that higher-order quantifiers that require an additional processing step induce higher activation in Broca’s region than first-order quantifiers. In other words, increasing *processing* demands, be they the resolution of an implicit negation in a negative quantifier or the additional semantic computation in a higher-order quantifier, are associated with stronger recruitment of Broca’s region. Given this pattern, one might be tempted to speculate that it is the semantic *evaluation* rather than the semantic *representation* of quantifiers that is impaired in the PWNFA. This hypothesis would be commensurate with the observation that the PWNFA in the present study seemed to have selective impairments in processing the negative quantifier “few,” for which the curves of the acceptability ratings should have been some kind of mirror image of those for “many” – rather than running roughly in parallel.

One other, linguistic, aspect in context of semantic processing needs consideration. As outlined in the introduction, the “weak” quantifiers “many” and “few” not only allow testing semantic flexibility, but they can also be processed either as proportional or as majority quantifiers. In the studies by Oaksford et al. (2002), Heim et al. (2012), or Pezzelle et al. (2018), the judgment of” few” and” many” crucially depended on the reference quantity, in this case the quantity of dots of the complement set the color of which had not been mentioned in the stimulus sentence. On the other hand, in the studies by Heim et al. (2015, 2020a,b) and Shikhare et al. (2015), “many” and “few” could be alternatively used as quasi-polar opposites, and thus, as majority quantifiers. However, it should be noted that the participants never had to decide directly between the two quantifiers, e.g., in a multiple choice setting. Instead, as in the studies by, e.g., Oaksford et al. (2002) and Pezzelle et al. (2018), each individual trial required evaluation whether the mentioned quantifier (out of two, or, in the 2012 study, out of six) appropriately described one particular scene of blue and yellow dots. This makes it more likely that “many” and “few” were also in these experiments, and therefore, also in the present one, processed as proportional rather than majority quantifiers.

Furthermore, one might wonder whether not a problem in the Semantic Evaluation but in one of the preceding processes (Estimation, Comparison, related to visual approximation of the magnitude of the target and complement set) was the reason for the observed difficulties for the processing of the negative quantifier “few” in PWNFA. Since both processes rely on posterior regions in particular in the IPS and IPL which are likely damaged in PWNFA, such impairments would indirectly also result in impediments in the subsequent semantic evaluation and truth value judgment. We cannot fully rule out this explanation. It is, however, not very likely, for two reasons. First, as reported in the Introduction, Estimation and Comparison are supported by bilateral regions in the IPL and IPS, i.e., also in the unimpaired hemisphere, whereas semantic evaluation relies exclusively on frontal areas in the left hemisphere where damage has thus much more grave consequences. Second, the processes of Estimation and Comparison are identical for both quantifiers and groups, since the same picture set with the same proportions of blue and yellow circles were presented. If a visual/perceptual or numerosity-related process was the reason for the worse performance in PWNFA, this should occur for both quantifiers, not just for “few,” as was the case in our results. Thus, the explanation of a semantic deficit has, in our view, a higher plausibility.

Finally, there is the possibility that the patients have a general cognitive or language deficit. With the inclusion criteria and the initial trial run before the actual experiments, we tried to exclude this option right from the start. The data we obtained from the participants further speak against this option. Since the non-fluent patients were not below PR = 16 in Corsi and NVLT (see Table 1), a general cognition deficit cannot likely be assumed. A “general language” deficit is also rather unlikely, as the accuracy in Block 1 for “many” at extreme proportions was very high, which would not be possible if one assumes a general language deficit undermining all evaluation processes.

### Semantic flexibility in quantifier processing in aphasia: feedback vs. adaptation

The next question is why feedback succeeded to cause a semantic shift in PWFA, but adaptation did not? The combined analysis shows that the experiment, i.e., the choice of learning method, is indeed relevant for the occurrence of a shift in PWFA. However, this does not yet clarify the cause of why feedback is effective, but adaptation is not. In neurotypical participants both methods successfully shifted the meaning of both quantifiers (Heim et al., 2015, 2016, 2020b). This indicates that the absence of a shift, the lack of adaptation is connected to aphasia, respectively, the lesion causing it.

One could suspect that explicit reinforcement techniques involving external feedback provide a stronger learning impulse than a subtle change of stimuli range that must be noticed subconsciously. Adaptation in the context of this experimental paradigm requires implicit learning. This means “learning without awareness” or learning “without a conscious intention” (Seger, 1994; Schuchard and Thompson, 2014), as it may happen in natural language use in dialogs (Pezzelle and Fernández, 2023). In this case, learning to call a proportion of 40% of colored circles “many.” The participants were not informed in advance that the stimuli selection would be restricted in the second block or that it would be tested to what extent this would influence their evaluation behavior. Since each trial lasted only about 6 s, there was extremely little to no time to consciously think about an overarching pattern leaving only room for subconscious learning. Adaptation as a more subtle form of learning might be more likely to be impaired in the case of a cognitive processing disorder, e.g., due to a lesion after stroke. After all, feedback offers the possibility to consciously react to it and to actively change evaluation behavior. Previous studies on implicit learning in PWA yielded inconsistent results on this subject. Vadinova et al. (2020) found that PWA with frontal and posterior lesions showed impairments in “implicit statistical learning (ISL).” This matches our results in that neither PWFA nor PWNFA, patients with probably differently localized lesions (Benson, 1967; Cipolotti et al., 2021) achieved a semantic shift by adaptation. But Vadinova et al. (2020) also noted that ISL is “not completely absent” but still possible to a limited extent. Schuchard and Thompson (2014) even demonstrated in their study that implicit learning in PWA is less impaired than explicit learning, which they suggest causes extra load on working memory because it requires the additional processing of feedback. Schuchard et al. (2017) partly supported these findings which appear to contradict our results. Nevertheless, it is possible that participants of our study also possessed limited implicit learning abilities, but that these were insufficient to achieve adaptation in this setting. In line with this thought, Schuchard et al. (2017) also stated that implicit learning is possible but not always successful. Overall, the study situation remains inconclusive and ambiguous and subject to debate. Our findings may inspire further studies to examine learning capacities of PWFA and PWNFA in more detail. These may also include different task settings, as Bremnes et al. (2022) demonstrated differential brain responses for verification vs. comprehension tasks.

### Task difficulty and participants vs. non-participants

Some PWA passed the initial screening for participation, while others failed to show sufficiently good performance. Thus, there seems to be substantial (and perhaps systematic) variability in PWAs’ abilities for quantifier processing. Interestingly, the polarity effect (i.e., higher processing demands for negative quantifiers, reflected in longer RTs), which is a robust finding in neurotypical persons (Heim et al., 2012, 2020b; Agmon et al., 2019, 2021) was also found in both experiments of the present study (i.e., the main effect of QUANTIFIER). This indicates that those participants who passed the initial screening had no apparent deficits in judging quantified statements in general even in the case of increased processing cost associated with the implicit negation in “few” (Just and Carpenter, 1971; Deschamps et al., 2015; Shikhare et al., 2015; Heim et al., 2020b; Agmon et al., 2021; Grodzinsky et al., 2021). Note that the in-depth analysis of the Polarity effect, i.e., the separate analysis for the two sub-groups, again demonstrated that the PWFA had a more consistent effect than the group of PWNFA. The GROUP × QUANTIFIER interaction results from the difference in acceptance at the critical proportion between quantifiers. This difference in acceptability is more pronounced for PWFA, who demonstrate very different acceptance levels of “few” and “many” at 40%, and less pronounced for PWFNA, which accept “few” and “many” at 40% with almost similar frequency. As briefly described in the discussion of Experiment 1, we interpret this as an attenuated polarity effect in PWNFA, i.e., PWNFA cannot process the negative quantifier as well or differentiate it from the positive quantifier, resulting in similar acceptance levels at the same proportion (see Figure 4). Thus, fluency of aphasia seems to play some relevant role, both for quantifier processing in general and also for the semantic flexibility.

To identify potential causes for non-participation, we compared some characteristics (fluency and performance in clinical language tests) between the two groups (participants and non-participants). Non-participants performed significantly worse in the Token Test and language comprehension test (cf. Figure 7) of the AAT indicating a generally higher level of severity of aphasia and worse semantic processing (Willmes et al., 1983). Thus, at least in some respects the linguistic abilities of non-participants appear to be significantly worse than those of the participants. As indicated in the introduction, working memory could be an important limiting factor. Since majority quantifiers place disproportionately higher demands on working memory and previous studies have repeatedly found that aphasia patients have impairments in working memory (Caspari et al., 1998; Friedmann and Gvion, 2003; Wright and Shisler, 2005; Sung et al., 2009; Christensen and Wright, 2010; Potagas et al., 2011; Mayer and Murray, 2012; Wright and Fergadiotis, 2012), it is expected that processing these quantifiers relates to difficulties, especially when it comes to equivocal sentence-picture pairs which related to higher processing cost in previous studies (McMillan et al., 2013).

As described above (Table 1), the non-verbal memory performance in the Corsi block tapping test showed that two PWA have a critical percentile rank (PR) but in the NVLT the PR is within normal range. Conversely, four PWA have an abnormal PR in the NVLT, but the Corsi is normal. In the TAP (Go-NoGo), all percentile ranks are in the average range or above. We therefore argue that the brain damage of PWA in this study does not cause global cognitive or non-verbal working memory problems, but only very isolated abnormalities. This is consistent with the fact that the positive quantifier “many” could be processed almost as well by PWA as by healthy people, even though it also classifies as a majority quantifier.

## Limitations

Our conclusions are limited by the absence of imaging data, i.e., information on lesion location and size. Functional imaging studies are strongly recommended to further investigate and validate relevant anatomical areas in more detail and relate lesion location, regions for speech fluency and quantifier processing. In addition, it would be advisable to collect more data on PWNFA. Because fewer patients who qualified for participation in this study were diagnosed with non-fluent aphasia, the results might be less sustainable and would benefit from a larger sample size. Since we only used the two quantifiers “many” and “few,” it also remains unclear whether and how other quantifiers would be processed by PWA. Investigating processing of other quantifier types in PWA could provide interesting additional insights. Maybe quantifiers containing numbers thus providing a specific external reference could be easier processed compared to more “fuzzy” quantifiers with primarily internal reference. Moreover, it may be useful to further investigate whether or to what extent implicit learning is limited compared to explicit learning in PWA, more specifically, comparing learning success between PWFA and PWNFA.

We tested here the result of a sequence of more than three processing steps (Heim et al., 2012) and see different outcomes. From our work, the individual processing steps cannot be traced. Consequently, it is also not possible to determine which processing step does not work for whom. For this, further studies are needed, designed to capture the individual steps of processing.

Finally, there was no control group included in this study, so no direct comparison of the size of the semantic flexibility effect in PWFA and neurotypical people can be made. However, the primary goal of this first study of the effect in PWA was to see whether at all, and if so, in which paradigm there would be statistically significant adaptation of the internal criterion. In subsequent studies, one can now focus on the feedback paradigm from Experiment 2, extend the setting by a neurotypical matched control group, and also acquire neurophysiological or hemodynamic data from both groups.

## Conclusion

This study examined quantifier processing in patients with aphasia. The results demonstrate varying degrees of impairment. While some patients were unable to participate, others performed well with response patterns similar to neurotypical individuals. The polarity effect is noticeable in RT data and acceptability judgments. Especially PWNFA showed clear difficulties in the evaluation of the negative quantifier and failed to achieve a semantic shift in both experiments. In contrast, a shift could be induced in PWFA, but only by feedback, not by adaptation. It appears that implicit learning may be impaired in PWA. Moreover, since PWFA and PWNFA show strikingly different performance levels there seems to be a link between fluency of speech and quantifier processing. Further studies are recommended to explore these connections.

## Data availability statement

The datasets generated for this study are available on request to the corresponding author.

## Ethics statement

The experiments were approved by the ethics committee of RWTH Aachen University (EK 391/21). Informed written consent was obtained from all participants. Capability to consent was verified by the supervising senior physician.

## Author contributions

BR: Data curation, Formal analysis, Investigation, Methodology, Visualization, Writing – original draft, Writing – review & editing. WG: Investigation, Methodology, Writing – review & editing. NP: Methodology, Software, Writing – review & editing. JP: Methodology, Resources, Writing – review & editing. KH: Methodology, Resources, Writing – review & editing. CW: Conceptualization, Project administration, Writing – review & editing. SH: Conceptualization, Data curation, Methodology, Project administration, Resources, Supervision, Writing – review & editing.
