# Supplementary material for: Quantifier processing and semantic flexibility in patients with aphasia
Source: Front Psychol. 2024 Jul 18;15:1328853. doi: 10.3389/fpsyg.2024.1328853 (PMC11294751; doi:10.3389/fpsyg.2024.1328853)
Supplement: Supplementary file 1 [file Data_Sheet_1.pdf]

Appendix

Appendix A1: Full set of parameters for the LMM analysis of RTs for Experiment 1

						95% CI	
Parameter	Estimate	SEM	df	t	p	Lower	Upper
Constant Term	2008.033333	134.477797	3156.000	14.932	.000	1744.360573	2271.706094
GROUP	-630.791954	155.949466	3156.000	-4.045	.000	-936.564557	-325.019351
BLOCK	-300.133333	190.180325	3156.000	-1.578	.115	-673.022927	72.756261
QUANTIFIER	-8.613978	188.640378	3156.000	-.046	.964	-378.484174	361.256217
GROUP × BLOCK	355.259770	220.545850	3156	1.611	.107	-77.167992	787.687532
GROUP × QUANTIFIER	360.554417	219.057629	3156.000	1.646	.100	-68.955367	790.064202
BLOCK × QUANTIFIER	453.920875	269.030377	3156.000	1.687	.092	-73.571273	981.413023
GROUP × BLOCK × QUANTIFIER	-305.950060	311.966061	3156.000	-.981	.327	-917.626889	305.726769
[PROPORTION=20]	-124.885185	195.391710	3156	-.639	.523	-507.992824	258.222454
[PROPORTION=30]	29.395238	193.546610	3156	.152	.879	-350.094684	408.885160
[PROPORTION=40]	85.735897	197.359450	3156	.434	.664	-301.229922	472.701717
[PROPORTION=50]	239.600000	190.180325	3156.000	1.260	.208	-133.289594	612.489594
[PROPORTION=60]	-43.343678	191.812804	3156	-.226	.821	-419.434100	332.746743
[PROPORTION=70]	-100.783333	187.185172	3156	-.538	.590	-467.800283	266.233616
[PROPORTION=80]	0[b]	0	.	.	.	.	.
GROUP × [PROPORTION=20]	412.896679	225.055184	3156.000	1.835	.067	-28.372607	854.165966
GROUP × [PROPORTION=30]	198.556564	223.296535	3156	.889	.374	-239.264512	636.377640
GROUP × [PROPORTION=40]	315.999735	226.765668	3156.000	1.394	.164	-128.623326	760.622795
GROUP × [PROPORTION=50]	345.599097	221.050186	3156.000	1.563	.118	-87.817525	779.015719
GROUP × [PROPORTION=60]	400.613927	222.118389	3156.000	1.804	.071	-34.897139	836.124992
GROUP × [PROPORTION=70]	194.093678	217.968356	3156	.890	.373	-233.280352	621.467709
BLOCK × [PROPORTION=20]	768.630346	271.593653	3156	2.830	.005	236.112343	1301.148350
BLOCK × [PROPORTION=30]	478.642262	269.255601	3156.000	1.778	.076	-49.291486	1006.576010
BLOCK × [PROPORTION=40]	541.401140	277.720494	3156.000	1.949	.051	-3.129859	1085.932138

BLOCK × [PROPORTION=50]	197.153846	274.079019	3156.000	.719	.472	-340.237254	734.544946
BLOCK × [PROPORTION=60]	485.015107	272.493012	3156.000	1.780	.075	-49.266286	1019.296499
BLOCK × [PROPORTION=70]	268.607471	268.012015	3156.000	1.002	.316	-256.887958	794.102901
QUANTIFIER × [PROPORTION=20]	-56.534170	272.739268	3156.000	-.207	.836	-591.298398	478.230059
QUANTIFIER × [PROPORTION=30]	-365.054593	276.879645	3156.000	-1.318	.187	-907.936926	177.827740
QUANTIFIER × [PROPORTION=40]	67.987605	275.368260	3156.000	.247	.805	-471.931332	607.906542
QUANTIFIER × [PROPORTION=50]	-187.259355	274.537072	3156.000	-.682	.495	-725.548567	351.029857
QUANTIFIER × [PROPORTION=60]	136.995752	271.420482	3156.000	.505	.614	-395.182713	669.174217
QUANTIFIER × [PROPORTION=70]	144.963978	265.750787	3156.000	.545	.585	-376.097825	666.025782
GROUP × BLOCK × [PROPORTION=20]	-714.033186	314.410039	3156	-2.271	.023	-1330.501960	-97.564412
GROUP × BLOCK × [PROPORTION=30]	-434.007858	312.044143	3156	-1.391	.164	-1045.837782	177.822067
GROUP × BLOCK × [PROPORTION=40]	-410.056312	319.487878	3156.000	-1.283	.199	-1036.481287	216.368663
GROUP × BLOCK × [PROPORTION=50]	-228.523085	316.793883	3156	-.721	.471	-849.665899	392.619730
GROUP × BLOCK × [PROPORTION=60]	-537.978753	315.184542	3156.000	-1.707	.088	-1155.966108	80.008603
GROUP × BLOCK × [PROPORTION=70]	-269.251541	310.971717	3156.000	-.866	.387	-878.978743	340.475661
GROUP × QUANTIFIER × [PROPORTION=20]	-353.647649	315.055037	3156	-1.122	.262	-971.381082	264.085785
GROUP × QUANTIFIER × [PROPORTION=30]	97.341427	318.423586	3156.000	.306	.760	-526.996773	721.679627
GROUP × QUANTIFIER × [PROPORTION=40]	-342.622702	317.564758	3156.000	-1.079	.281	-965.276985	280.031581
GROUP × QUANTIFIER × [PROPORTION=50]	59.545106	317.692001	3156	.187	.851	-563.358664	682.448877
GROUP × QUANTIFIER × [PROPORTION=60]	-232.153701	314.263084	3156.000	-.739	.460	-848.334339	384.026937
GROUP × QUANTIFIER × [PROPORTION=70]	-29.107304	309.142298	3156.000	-.094	.925	-635.247535	577.032926
BLOCK × QUANTIFIER × [PROPORTION=20]	-547.317888	383.097966	3156.000	-1.429	.153	-1298.464177	203.828400

BLOCK × QUANTIFIER × [PROPORTION=30]	-14.823137	385.347607	3156	-.038	.969	-770.380331	740.734057
BLOCK × QUANTIFIER × [PROPORTION=40]	-680.405613	390.905506	3156	-1.741	.082	-1446.860269	86.049044
BLOCK × QUANTIFIER × [PROPORTION=50]	-54.355234	392.295430	3156	-.139	.890	-823.535136	714.824668
BLOCK × QUANTIFIER × [PROPORTION=60]	-596.577781	387.209096	3156	-1.541	.123	-1355.784827	162.629265
BLOCK × QUANTIFIER × [PROPORTION=70]	-342.637870	381.443991	3156.000	-.898	.369	-1090.541183	405.265442
GROUP × BLOCK × QUANTIFIER × [PROPORTION=20]	309.691528	443.541687	3156	.698	.485	-559.967728	1179.350783
GROUP × BLOCK × QUANTIFIER × [PROPORTION=30]	-185.662307	445.491948	3156.000	-.417	.677	-1059.145470	687.820856
GROUP × BLOCK × QUANTIFIER × [PROPORTION=40]	509.784629	450.381122	3156.000	1.132	.258	-373.284815	1392.854072
GROUP × BLOCK × QUANTIFIER × [PROPORTION=50]	-56.874740	452.425564	3156.000	-.126	.900	-943.952754	830.203274
GROUP × BLOCK × QUANTIFIER × [PROPORTION=60]	399.103546	447.419777	3156	.892	.372	-478.159543	1276.366634
GROUP × BLOCK × QUANTIFIER × [PROPORTION=70]	288.928875	442.111964	3156.000	.654	.513	-577.927100	1155.784849

Appendix A2: ANOVA for Acceptability Ratings for Experiment 1

In line with the analysis of the previous studies (Heim et al. 2015; 2016; 2020a,b), the participants' acceptability judgments of the presented quantifier at certain proportions were first aggregated per participant, quantifier, experimental block, and proportion of circles in the target color. Then they were aggregated a second time for the latter three factors obtaining data for the descriptive analyses. The aggregated values were subjected to a 2 × 2 ANOVA with factors QUANTIFIER (many/few)

and BLOCK (baseline/test). Factors were examined at proportion 40% at which a change in acceptability due to adaptation was most likely to occur. In this way, the data of the whole group as well as that of PWFA and PWNFA were analyzed.

The 2 × 2 ANOVA for the entire group revealed a significant main effect for QUANTIFIER ($F(1,14) = 20.194$; $p < 0.001$), whereas the main effect BLOCK ($F(1,14) = 0.267$; $p = 0.614$) and the interaction (QUANTIFIER × BLOCK: $F(1,14) = 0.444$; $p = 0.516$) were not significant. The identically designed ANOVAs for PWFA and PWNFA yielded the following results: PWFA showed a significant main effect QUANTIFIER ($F(1,10) = 36.352$; $p < 0.001$) and non-significant main effect BLOCK ($F(1,10) = 0.008$; $p = 0.929$) and interaction (QUANTIFIER × BLOCK: $F(1,10) = 1.290$; $p = 0.283$). For PWNFA, all three results were non-significant (QUANTIFIER: $F(1,3) = 0.232$; $p = 0.663$; BLOCK: $F(1,3) = 4.654$; $p = 0.120$; interaction QUANTIFIER × BLOCK: $F(1,3) = 0.944$; $p = 0.403$). Hence no adaptation occurred in all three groups: all PWA and the subgroups PWFA and PWNFA (cf. Figure 4). Neither the criterion for “many” nor that for “few” could be shifted. Experiment 1 thus shows no evidence of semantic flexibility in PWA.

Appendix B1: Full set of parameters for the LMM analysis of RTs for Experiment 2

						95% CI	
Parameter	Estimate	SEM	df	t	p	Lower	Upper
Constant Term	1714.166667	103.240427	4715	16.604	.000	1511.767192	1916.566141
GROUP	-331.575758	118.532501	4715.000	-2.797	.005	-563.954844	-99.196671
BLOCK	353.023810	146.004012	4715	2.418	.016	66.787727	639.259892
QUANTIFIER	264.961538	148.785290	4715.000	1.781	.075	-26.727150	556.650227

GROUP × BLOCK	-436.167749	167.630271	4715.000	-2.602	.009	-764.801404	-107.534093
GROUP × QUANTIFIER	163.742126	170.289953	4715.000	.962	.336	-170.105748	497.590000
BLOCK × QUANTIFIER	-217.890110	208.456792	4715.000	-1.045	.296	-626.562822	190.782602
GROUP × BLOCK × QUANTIFIER	178.307657	238.953083	4715.000	.746	.456	-290.152034	646.767349
[PROPORTION=20]	88.190476	146.004012	4715.000	.604	.546	-198.045606	374.426558
[PROPORTION=30]	169.883333	147.817796	4715	1.149	.251	-119.908613	459.675280
[PROPORTION=40]	486.083333	151.965793	4715	3.199	.001	188.159373	784.007294
[PROPORTION=50]	151.345528	146.891582	4715.000	1.030	.303	-136.630607	439.321664
[PROPORTION=60]	219.443089	146.891582	4715	1.494	.135	-68.533046	507.419225
[PROPORTION=70]	-124.532520	146.891582	4715	-.848	.397	-412.508656	163.443615
GROUP × [PROPORTION=20]	288.562126	167.707472	4715	1.721	.085	-40.222879	617.347131
GROUP × [PROPORTION=30]	163.792933	169.288881	4715.000	.968	.333	-168.092374	495.678240
GROUP × [PROPORTION=40]	98.056527	172.998594	4715.000	.567	.571	-241.101551	437.214604
GROUP × [PROPORTION=50]	407.863562	168.558738	4715.000	2.420	.016	77.409678	738.317447
GROUP × [PROPORTION=60]	210.897299	168.480745	4715.000	1.252	.211	-119.403683	541.198282
GROUP × [PROPORTION=70]	183.262222	168.480745	4715.000	1.088	.277	-147.038761	513.563205

BLOCK × [PROPORTION=20]	146.547619	206.480853	4715.000	.710	.478	-258.251331	551.346569
BLOCK × [PROPORTION=30]	154.003114	209.731169	4715	.734	.463	-257.167974	565.174201
BLOCK × [PROPORTION=40]	-152.073810	211.999300	4715	-.717	.473	-567.691492	263.543873
BLOCK × [PROPORTION=50]	161.694765	209.079410	4715.000	.773	.439	-248.198569	571.588099
BLOCK × [PROPORTION=60]	-418.292102	207.736068	4715.000	-2.014	.044	-825.551858	-11.032346
BLOCK × [PROPORTION=70]	75.528091	206.510131	4715.000	.366	.715	-329.328257	480.384438
QUANTIFIER × [PROPORTION=20]	-132.093681	209.731169	4715	-.630	.529	-543.264769	279.077406
QUANTIFIER × [PROPORTION=30]	-384.678205	209.731169	4715.000	-1.834	.067	-795.849292	26.492882
QUANTIFIER × [PROPORTION=40]	-530.836538	213.924272	4715	-2.481	.013	-950.228067	-111.445010
QUANTIFIER × [PROPORTION=50]	-228.863977	209.700179	4715	-1.091	.275	-639.974309	182.246354
QUANTIFIER × [PROPORTION=60]	-29.737961	209.079410	4715.000	-.142	.887	-439.631295	380.155373
QUANTIFIER × [PROPORTION=70]	298.867730	209.700179	4715	1.425	.154	-112.242602	709.978062

GROUP × BLOCK × [PROPORTION=20]	-122.254883	237.229591	4715	-.515	.606	-587.335725	342.825959
GROUP × BLOCK × [PROPORTION=30]	-176.118774	239.955255	4715.000	-.734	.463	-646.543191	294.305643
GROUP × BLOCK × [PROPORTION=40]	111.335465	241.994526	4715	.460	.645	-363.086877	585.757806
GROUP × BLOCK × [PROPORTION=50]	-13.800438	239.605334	4715.000	-.058	.954	-483.538847	455.937972
GROUP × BLOCK × [PROPORTION=60]	476.451309	238.267755	4715	2.000	.046	9.335179	943.567438
GROUP × BLOCK × [PROPORTION=70]	-21.826899	237.199670	4715.000	-.092	.927	-486.849083	443.195285
GROUP × QUANTIFIER × [PROPORTION=20]	-348.108533	240.283712	4715	-1.449	.147	-819.176881	122.959814
GROUP × QUANTIFIER × [PROPORTION=30]	23.615057	240.228159	4715	.098	.922	-447.344379	494.574494
GROUP × QUANTIFIER × [PROPORTION=40]	154.323980	244.061654	4715	.632	.527	-324.150899	632.798859
GROUP × QUANTIFIER × [PROPORTION=50]	69.541415	240.425040	4715	.289	.772	-401.804001	540.886832
GROUP × QUANTIFIER × [PROPORTION=60]	-166.819482	239.715036	4715	-.696	.487	-636.772958	303.133995
GROUP × QUANTIFIER × [PROPORTION=70]	-245.227883	240.146379	4715	-1.021	.307	-716.026993	225.571227

BLOCK × QUANTIFIER × [PROPORTION=20]	58.898559	294.756631	4715	.200	.842	-518.962161	636.759280
BLOCK × QUANTIFIER × [PROPORTION=30]	340.640965	298.693536	4715	1.140	.254	-244.937928	926.219857
BLOCK × QUANTIFIER × [PROPORTION=40]	593.781326	300.618056	4715.000	1.975	.048	4.429474	1183.133179
BLOCK × QUANTIFIER × [PROPORTION=50]	336.986780	296.582751	4715.000	1.136	.256	-244.453989	918.427549
BLOCK × QUANTIFIER × [PROPORTION=60]	454.352096	296.730277	4715	1.531	.126	-127.377892	1036.082085
BLOCK × QUANTIFIER × [PROPORTION=70]	-273.264094	296.878872	4715	-.920	.357	-855.285398	308.757211
GROUP × BLOCK × QUANTIFIER × [PROPORTION=20]	-149.126658	338.044533	4715	-.441	.659	-811.851893	513.598577
GROUP × BLOCK × QUANTIFIER × [PROPORTION=30]	-273.775307	341.367242	4715.000	-.802	.423	-943.014604	395.463990
GROUP × BLOCK × QUANTIFIER × [PROPORTION=40]	-480.530772	343.207460	4715	-1.400	.162	-1153.377754	192.316210
GROUP × BLOCK × QUANTIFIER × [PROPORTION=50]	-321.546755	339.914232	4715.000	-.946	.344	-987.937473	344.843964

GROUP × BLOCK × QUANTIFIER × [PROPORTION=60]	-338.778461	339.728135	4715	-.997	.319	-1004.804341	327.247420
GROUP × BLOCK × QUANTIFIER × [PROPORTION=70]	204.361606	339.857931	4715	.601	.548	-461.918735	870.641947

Appendix B2: ANOVA for Acceptability Ratings for Experiment 2

For Experiment 2 we proceeded with the analysis in the same way as for Experiment 1.

The 2 × 2 ANOVA regarding acceptability in Experiment 2 yielded different results depending on participant group. The analysis of the whole group showed similar results to Experiment 1, i.e. only the main effect QUANTIFIER ($F(1,14) = 27.440$; $p < 0.001$) reached significance, whereas main effect BLOCK ($F(1,14) = 2.055$; $p = 0.174$) and the interaction ($F(1,14) = 2.911$; $p = 0.110$) were not significant. PWNFA demonstrated no significant interaction either (QUANTIFIER × BLOCK: $F(1,3) = 0.311$; $p = 0.616$), a non-significant main effect BLOCK ($F(1,3) = 0.275$; $p = 0.636$) but also a non-significant main effect QUANTIFIER ($F(1,3) = 0.539$; $p = 0.516$).

In contrast, the ANOVA for PWFA revealed a significant interaction (QUANTIFIER × BLOCK: $F(1,10) = 9.819$; $p = 0.011$) in addition to a significant main effect QUANTIFIER ($F(1,10) = 56.063$; $p < 0.001$) and a non-significant main effect BLOCK ($F(1,10) = 2.351$, $p = 0.156$). As opposed to PWNFA, acceptability of quantifiers at 40% target color actually changed in PWFA.

Post-hoc t-tests showed that this change in acceptability ratings extended to both the positive quantifier “many” ($t(10) = -3.127$; $p = 0.005$ one-tailed) and the negative quantifier “few” ($t(10) = 1.865$; $p = 0.046$ one-tailed). The descriptive analysis confirms a shift in acceptability at proportion

40% in the trained direction from baseline block to test block (cf. Figure 6): The acceptability of "many" increased while that of "few" decreased.

Appendix C: Further analysis: Investigation of a relationship between experiment (learning method) and occurrence of a semantic shift.

In addition to the non-participant analysis, we also added another test regarding acceptability judgments. Because of a significant interaction in the 2 × 2 ANOVA of the PWFA in Experiment 2 (feedback) but a non-significant interaction in the very same group in Experiment 1 (adaptation) we wanted to investigate the influence of experiment choice. To assess this influence on the PWFA, we conducted a 2 × 2 × 2 ANOVA with factors QUANTIFIER, BLOCK and EXPERIMENT (Feedback/Adaptation). Besides main effect QUANTIFIER ($F(1,10) = 53.159$; $p = 0.001$) the three-way interaction was also significant (QUANTIFIER × BLOCK × EXPERIMENT: $F(1,10) = 8.570$; $p = 0.015$). Main effect BLOCK ($F(1,10) = 0.836$; $p = 0.382$) and EXPERIMENT ($F(1,10) = 0.263$; $p = 0.619$) as well as all two-way interactions were non-significant (QUANTIFIER × BLOCK: $F(1,10) = 0.863$; $p = 0.375$; QUANTIFIER × EXPERIMENT: $F(1,10) = 0.417$; $p = 0.533$; BLOCK × EXPERIMENT: $F(1,10) = 1.808$; $p = 0.208$). This means that for PWFA the type of experimental manipulation in Block 2, i.e., the type of learning method, was indeed relevant for the presence or absence of a semantic shift.
